# Design, Synthesis and Biological Evaluation of 4-Amino-*N*-(4-aminophenyl)benzamide Analogues of Quinoline-Based SGI-1027 as Inhibitors of DNA Methylation

**DOI:** 10.1002/cmdc.201300420

**Published:** 2014-02-13

**Authors:** Elodie Rilova, Alexandre Erdmann, Christina Gros, Véronique Masson, Yannick Aussagues, Valérie Poughon-Cassabois, Arumugam Rajavelu, Albert Jeltsch, Yoann Menon, Natacha Novosad, Jean-Marc Gregoire, Stéphane Vispé, Philippe Schambel, Fréderic Ausseil, François Sautel, Paola B Arimondo, Frédéric Cantagrel

**Affiliations:** [a] USR CNRS-Pierre Fabre No. 3388 ETaC, Centre de Recherche et de Développement Pierre Fabre (CRDPF)3 Ave Hubert Curien, 31035 Toulouse Cedex 01 (France) E-mail: paola.arimondo@etac.cnrs.frfrederic.cantagrel@etac.cnrs.fr; [b] Institute of Biochemistry, Faculty of Chemistry, University StuttgartPfaffenwaldring 55, 70569 Stuttgart (Germany); [c] Institut de Recherches Pierre Fabre17 Rue Jean Moulin, 81106 Castres Cedex (France)

**Keywords:** cytotoxicity, DNA methyltransferases, epigenetics, gene re-expression, inhibitors

## Abstract

Quinoline derivative SGI-1027 (*N*-(4-(2-amino-6-methylpyrimidin-4-ylamino)phenyl)-4-(quinolin-4-ylamino)benzamide) was first described in 2009 as a potent inhibitor of DNA methyltransferase (DNMT) 1, 3A and 3B. Based on molecular modeling studies, performed using the crystal structure of *Haemophilus haemolyticus* cytosine-5 DNA methyltransferase (MHhaI C5 DNMT), which suggested that the quinoline and the aminopyridimine moieties of SGI-1027 are important for interaction with the substrates and protein, we designed and synthesized 25 derivatives. Among them, four compounds—namely the derivatives **12**, **16**, **31** and **32**—exhibited activities comparable to that of the parent compound. Further evaluation revealed that these compounds were more potent against human DNMT3A than against human DNMT1 and induced the re-expression of a reporter gene, controlled by a methylated cytomegalovirus (CMV) promoter, in leukemia KG-1 cells. These compounds possessed cytotoxicity against leukemia KG-1 cells in the micromolar range, comparable with the cytotoxicity of the reference compound, SGI-1027. Structure–activity relationships were elucidated from the results. First, the presence of a methylene or carbonyl group to conjugate the quinoline moiety decreased the activity. Second, the size and nature of the aromatic or heterocycle subsitutents effects inhibition activity: tricyclic moieties, such as acridine, were found to decrease activity, while bicyclic substituents, such as quinoline, were well tolerated. The best combination was found to be a bicyclic substituent on one side of the compound, and a one-ring moiety on the other side. Finally, the orientation of the central amide bond was found to have little effect on the biological activity. This study provides new insights in to the structure–activity relationships of SGI-1027 and its derivative.

## Introduction

DNA methylation is an epigenetic modification that is involved in the control of gene expression.[1] It plays an important role in tumorigenesis and tumor maintenance.[2] Concomitantly to a global hypomethylation contributing to genetic instability, promoter hypermethylation of tumor suppressor genes is commonly observed in cancers, such that it can be used as a biomarker.[3] As with the other epigenetic marks, DNA methylation is reversible and constitutes a promising target in cancer treatment. DNA methylation is catalyzed by DNA methyltransferases (DNMT), which transfer the methyl group from the cofactor, *S*-adenosyl-l-methionine (AdoMet), to the position 5 of the deoxycytidine in a CpG context.[4] Two nucleosides analogues of cytidine, 5-azacytidine **2** (5aza) and 5-azadeoxycytidine **3** (5azadC), have been approved by the US Food and Drug Administration (FDA) for the treatment of myelodysplastic syndromes (MDS) and acute myeloide leukemia (AML).[5]

Beside nucleoside inhibitors, many efforts are made to develop new non-nucleoside inhibitors to overcome the limits of these azanucleosides, such as chemical instability and incorporation into DNA for activity.[6] In 2009, Datta et al. published a quinolone derivative, SGI-1027 (**1**), that depletes DNMT1.[7] Interestingly, its bisquaternary salt was first described for its antileukemic activity.[8] Inhibitory activity of SGI-1027 was described against DNMT1, 3A and 3B[7]. The compound was shown to degrade the enzymes, inhibit DNA methylation in colon cancer cell lines, and reactivate *P16*, *MLH1* and *TIMP3* genes.

Here, we describe the conception of new derivatives of SGI-1027 guided by a molecular modeling study. A total of 25 derivatives were synthetized and screened for their ability to inhibit the catalytic domain of human DNMT3A. Selectivity against several methyltransferases was assessed for the most potent inhibitors, as was their ability to reactivate gene expression in an epigenetic reporter system in a leukemia cell line.

## Results and Discussion

### Docking

**Figure 1:**
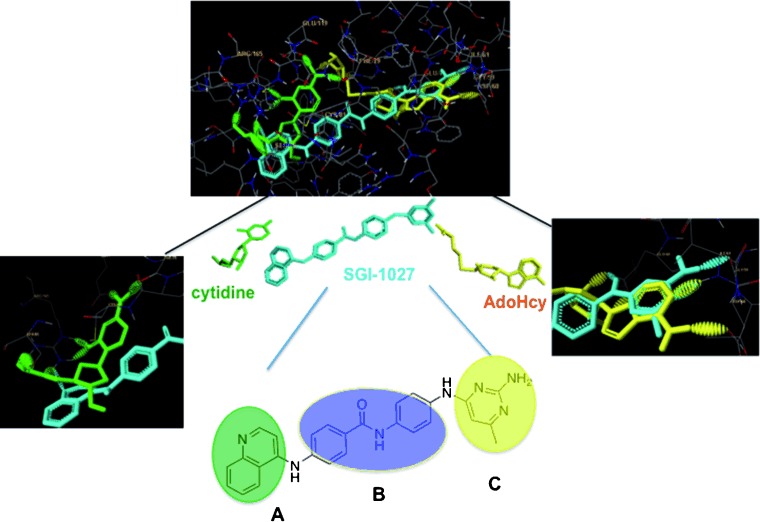
SGI-1027 molecular docking in *Haemophilus haemolyticus* cytosine-5 DNA methyltransferase (MHhaI C5 DNMT; PDB: 2HR1[16]). The co-crystalized *S*-adenosyl-l-homocysteine (AdoHcy) product of the methylation reaction is depicted in yellow and the cytidine substrate in green. The docked SGI-1027 is in cyan. The three parts of the molecule (A, B and C) explored here are encircled in colors in the zoom of SGI-1027.

To design new analogues of SGI-1027 and test their activity against the catalytic domain of hDNMT3A, we started by carrying out a docking study of SGI-1027 in the catalytic pocket of DNMTs. Recently, the crystal structure of the murine catalytic complex Dnmt3A/3L (PDB: 2QRV[9]) and several crystal structures of DNMT1 have been published (PDB: 3PTA,[10] 3OS5, 4DA4[11] and 3PT6[10]), together with molecular docking and pharmacophore modelling studies based on these structures.[12–14] Concerning the DNMT1 structures, we chose not to use them since the N-terminal domain of the C5 DNA methyltransferases is not well conserved and, in particular, DNMT1 contains an autoinhibition linker that is lacking in the DNMT3 isoforms[10, 11, 15] confering very specific properties to the interaction with the substrates and affecting inhibition, as observed for SGI-1027.[13, 14] Concerning the murine Dnmt3A catalytic domain co-crystallized with C-terminal Dnmt3L (PDB: 2QRV[9]), the substrate cytosine is not resolved in the crystal structure, only the cofactor (here the product *S*-adenosyl-l-homocysteine, AdoHcy). Interestingly, the DNMT3A catalytic pocket is well superimposable with that of the crystal structure of the bacterial *Haemophilus haemolyticus* cytosine-5 DNA methyltransferase (MHhaI C5 DNMT; PDB: 2HR1),[16] in particular for the AdoHcy molecule (shown in Figure S1 in the Supporting Information). We chose to conduct our docking studies on bacterial MHhaI C5 DNMT, since the catalytic pocket is well conserved among the C5 DNMTs and in the crystal structure of MHhaI C5 DNMT, both the co-factor (here the product AdoHcy) and the DNA substrate (deoxycytidine) are well resolved. Schematically, the catalytic pocket of the C5 DNA methyltransferases can be considered formed of three binding pockets (Figure 1): one pocket accomodates the adenine of AdoMet, another accomodates the amino acid of AdoMet, and the other accomodates the cytidine of the DNA that is flipped out of the DNA double helix into the catalytic pocket of the enzyme. Our docking studies of SGI-1027 (**1**) in MHhaI C5 DNMT (Figure 1) showed that the compound fits within the adenine binding pocket of the cofactor through the aminopyrimidine group (part C of SGI-1027) and within the cytidine binding pocket through the quinoline moiety (part A of SGI-1027). In our model, the orientation of the molecule seems to forbid any interaction with the amino acid binding pocket.

Noteworthy, the finding that part C fits into the adenine pocket of the cofactor is in agreement with the results obtained by induced-fit docking of SGI-1027 in the MTase domain of mDnmt3A,[14] even though part A is differently positioned compared with our docking result. In particular, the pyrimidine moiety (C) of SGI-1027 was found well superimposable to the six-membered ring of the adenine of AdoHcy (Figure 1, right-hand zoom). In addition, the amino group on the C4 of the adenine of AdoHcy formed hydrogen bonds with the carboxylic residue of Asp 60 (corresponding to Asp 682 in DNMT3A), and in parallel, the amino group of the pyrimidyl moiety of the inhibitor interacted with the amide residue of Asn 39 (corresponding to Ser 659 in DNMT3A). On the contrary, little interactions with the quinoline moiety A were observed in the cytosine pocket, except for some superimposition with the ribose of the cytidine. This can explain the differences observed for part A between our model and the induced-fit docking model of SGI-1027 in mDnmt3A described by Yoo et al.,[14] who used a crystal structure lacking the cytidine substrate in the resolution. Still, we found an interaction of part A with the enzyme, with Arg 165 (corresponding to Arg 788 in DNMT3A) (Figure 1, left-hand zoom). Based on these findings, we designed new derivatives of SGI-1027 to explore the substrate binding pockets guided by our docking model. More specifically, since few interactions were modelized for the quinoline moiety (part A, Figure 1), we began by modifying this part of the molecule aiming at improving the affinity of the compounds for the deoxycytidine binding pocket of DNMTs.

### Exploring the deoxycytidine pocket

**Scheme 1:**
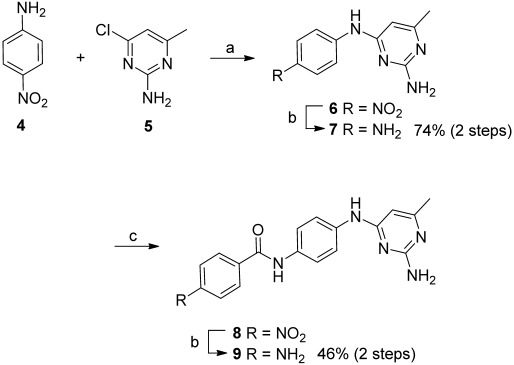
Synthesis of compound 9. *Reagents and conditions*: a) HCl, ethoxyethanol, reflux, 3 h; b) Fe, AcOH, EtOH/H_2_O (2:1), reflux, 15 h; c) 4-nitrobenzoyl chloride, Et_3_N, dioxane, 60 °C, 1 h.

**Scheme 2:**
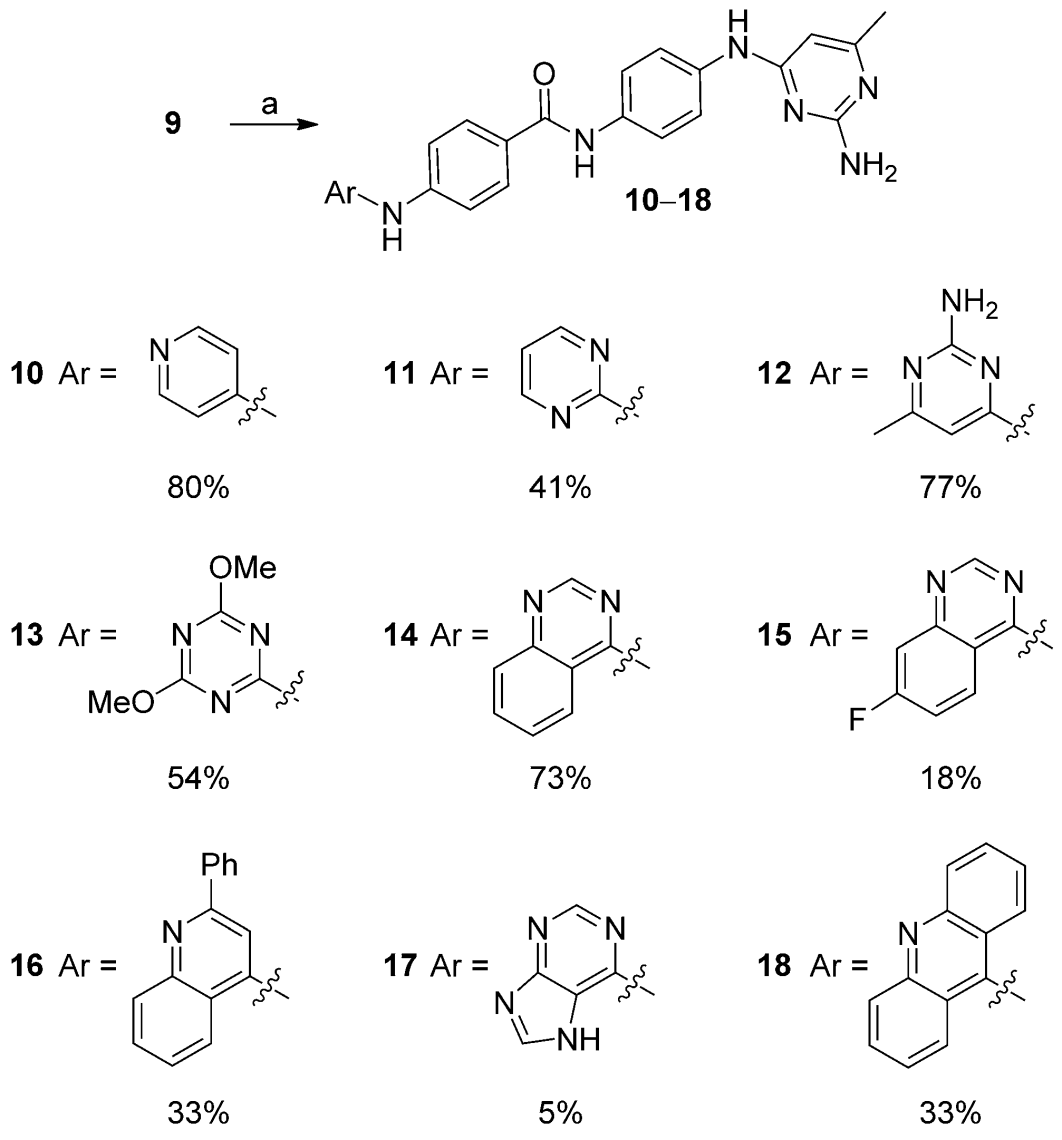
Synthesis of aryl derivatives. *Reagents and conditions*: a) ArCl, HCl (cat.), MeOH/H_2_O/EtOH (1:1:1), reflux, 22 h.

According to the above molecular docking studies, the quinoline moiety (Figure 1, ring A) is superimposable on the flipped-out deoxycytidine but lacks strong interactions in the pocket. Thus, its modifications can improve the affinity for the cytidine binding pocket. In a first series of compounds, this substituent was replaced with aromatic and heteroaromatic rings. The synthesis of compound **9** followed a published route (Scheme 1).[17] The desired derivatives were then obtained by direct reaction with miscellaneous aryl chlorides in the presence of catalytic amounts of acid (Scheme 2).

Yields were generally good, except upon use of chloropurine, because of its poor reactivity and the poor solubility of the final product (**17**) giving rise to material loss during purification. 4-Chloro-7-fluoroquinazoline—used to obtain **15**—was synthesized by reaction of *N*,*N*-dimethylformamide with 4-fluoro-2-aminobenzoic acid followed by intramolecular cyclization and chloration with thionyl chloride.[18]

**Figure 2:**
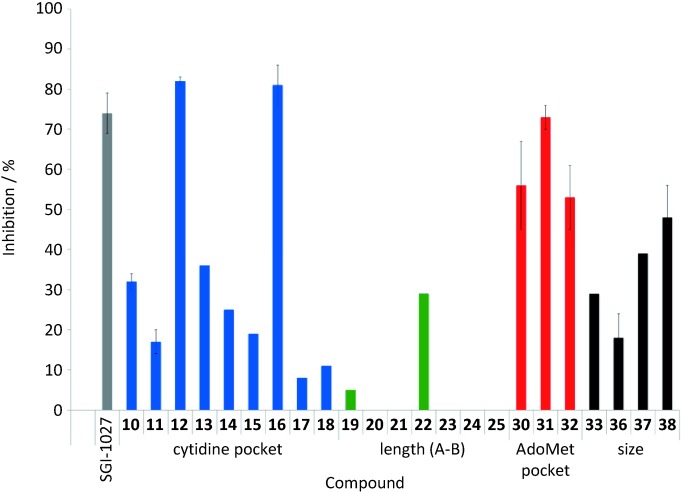
Inhibition of DNMT3A by parent compound SGI-1027 (▪) and synthesized compounds exploring the deoxycytidine pocket (▪), with varying tether length between the quinoline A and part B (▪), addressing the AdoMet pocket (▪), and with different moiety sizes addressing the two substrates pockets (▪). Percent inhibition was determined at a concentration of 10 μm.

The compounds were assayed for their ability to inhibit the catalytic activity of human DNMT3A at 32 and 10 μm (Table 1), with parent compound SGI-1027 as a reference. Compound **10**, where a pyridine replaces the quinoline of SGI-1027, inhibited 91 % of the enzyme activity at 32 μm, but this inhibition dramatically dropped at 10 μm (EC_50_ value of 13 μm±1 was calculated). Compounds **11** and **12**, bearing an unsubstituted pyrimidine and methylated aminopyrimidine, respectively, were slightly less active. Dimethoxy triazine **13** lost most of the inhibitory activity. In agreement with our model, the replacement of the quinoline moiety by bicyclic or tricyclic moieties was in general detrimental on the inhibition activity compared with the monocyclic substitution (**14**–**18**; Figure 2). In fact, the introduction of an adenine as ring A induced a strong decrease in the activity (analogue **17**), comforting the hypothesis that ring A resides in the cytidine pocket and not in the AdoMet pocket. In contrast, the addition of a phenyl group on the quinoline, analogue **16**, kept the activity at a good level, suggesting the presence of a hydrophobic pocket that might be worth exploring in future optimizations.

**Table 1:** Screening results for all compounds against DNMT3A catalytic activity

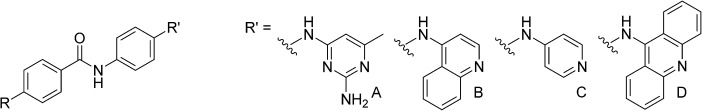									
	Compd		Inhibition [%]^[a]^			Compd		Inhibition [%]^[a]^	
	R	R′	32 μm	10 μm		R	R′	32 μm	10 μm
SGI-1027 (**1**)	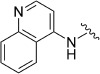	A	76±5	74±5	**20**	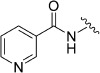	A	0^[b]^	0
**8**	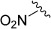	A	1^[b]^	n.d.	**21**	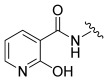	A	0^[b]^	0
**9**	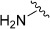	A	0^[b]^	n.d.	**22**	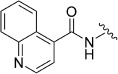	A	8	29
**10**	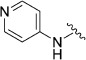	A	91±4	32	**23**	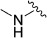	A	0^[b]^	0
**11**	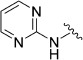	A	65±3	17	**24**	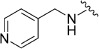	A	0^[b]^	0
**12**	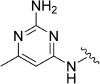	A	69±1	82±1	**25**	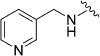	A	4	0
**13**	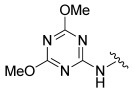	A	35	36	**30**	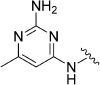	B	47±1	56±11
**14**	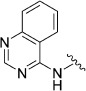	A	19	25	**31**	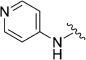	B	75±8	73±3
**15**	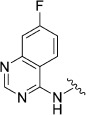	A	6	19	**32**	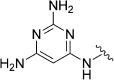	B	53±13	53±8
**16**	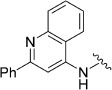	A	55±10	81±5	**33**	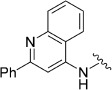	B	46	29
**17**	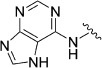	A	3^[b]^	8	**36**	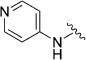	C	54±5	18
**18**	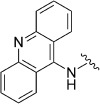	A	53±2	11	**37**	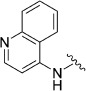	B	29	39
**19**	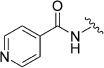	A	0^[b]^	5	**38**	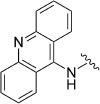	D	62±22	48±8

[a] When inhibition was >50 %, experiments were performed in triplicate/duplicate, and these data are reported as the mean±SEM. All other values are single determinations. Not determined (n.d.). [b] Inhibition was measured at 20 μm.

**Scheme 3:**
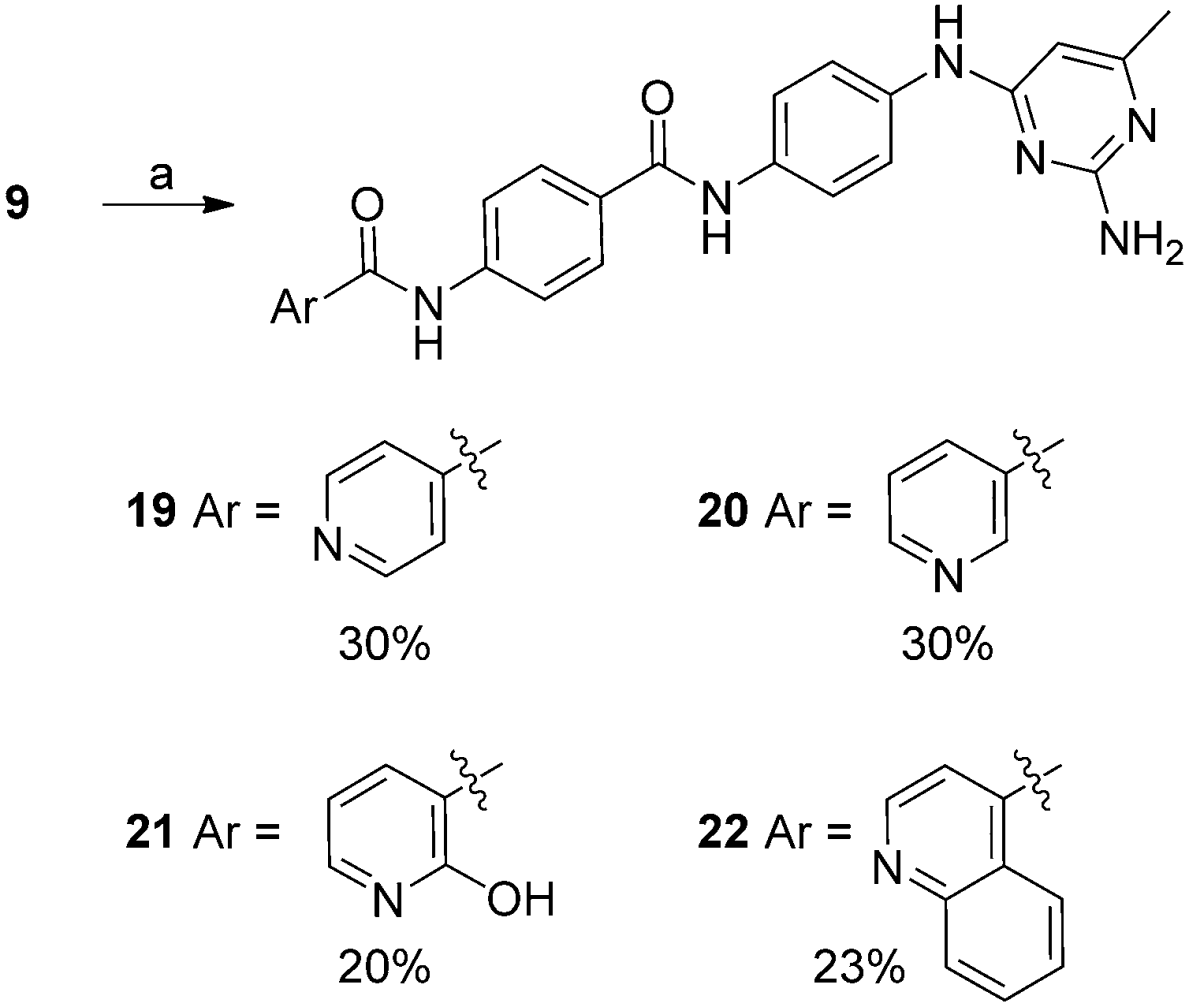
Synthesis of acyl derivatives. *Reagents and conditions*: a) Ar-C(O)-Cl, pyr., 0 °C→RT, o/n.

In a second series of derivatives, we studied the influence of the distance between rings A and B on the activity of the compounds. Preparation of compounds comprising an intercalated carbonyl group was easily achieved by acylation, albeit with only moderate yields (**19**–**22**; Scheme 3). Not only was the linker length changed in these compounds but also π-staking interactions and hydrogen bond counts were modified.

**Scheme 4:**
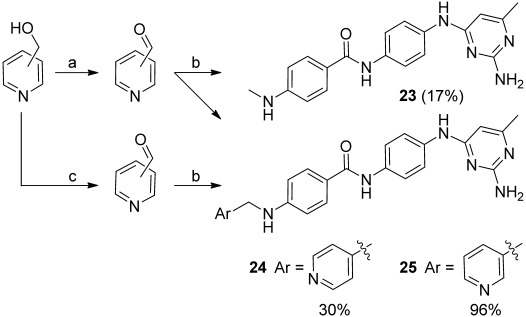
Synthesis of methylene derivatives. *Reagents and conditions*: a) PCC, CH_2_Cl_2_, RT, 90 min; b) 1. 9, MeOH, Et_3_N, RT, 3.5 h; 2. AcOH, NaBH_3_CN, RT, o/n; c) MnO_2_, CH_2_Cl_2_, RT, o/n.

Next, methylene groups were introduced. In the absence of a reaction with the corresponding chloromethylpyridines, methylene group introduction was performed by reductive amination (derivatives **23** and **24**; Scheme 4). Preparation of pyridine-3- or -4-carboxaldehyde from the corresponding alcohols could be done either with pyridinium chromate or manganese dioxide, but the latter was preferred because traces of oxidants led to formation of unexpected compound **25** that was used as a control (presumably via formaldehyde formation). All these modifications led to complete loss of activity (compounds **19**–**25**; Figure 2 and Table 1).

### Exploring the AdoMet pocket

A third series of products was devised to investigate the importance of the aminopyrimidine moiety (ring C in Figure 1). The synthetic pathway was similar to the one described above, but used 4-chloroquinoline instead of the chloropyrimidine as the starting materials.

**Scheme 5:**
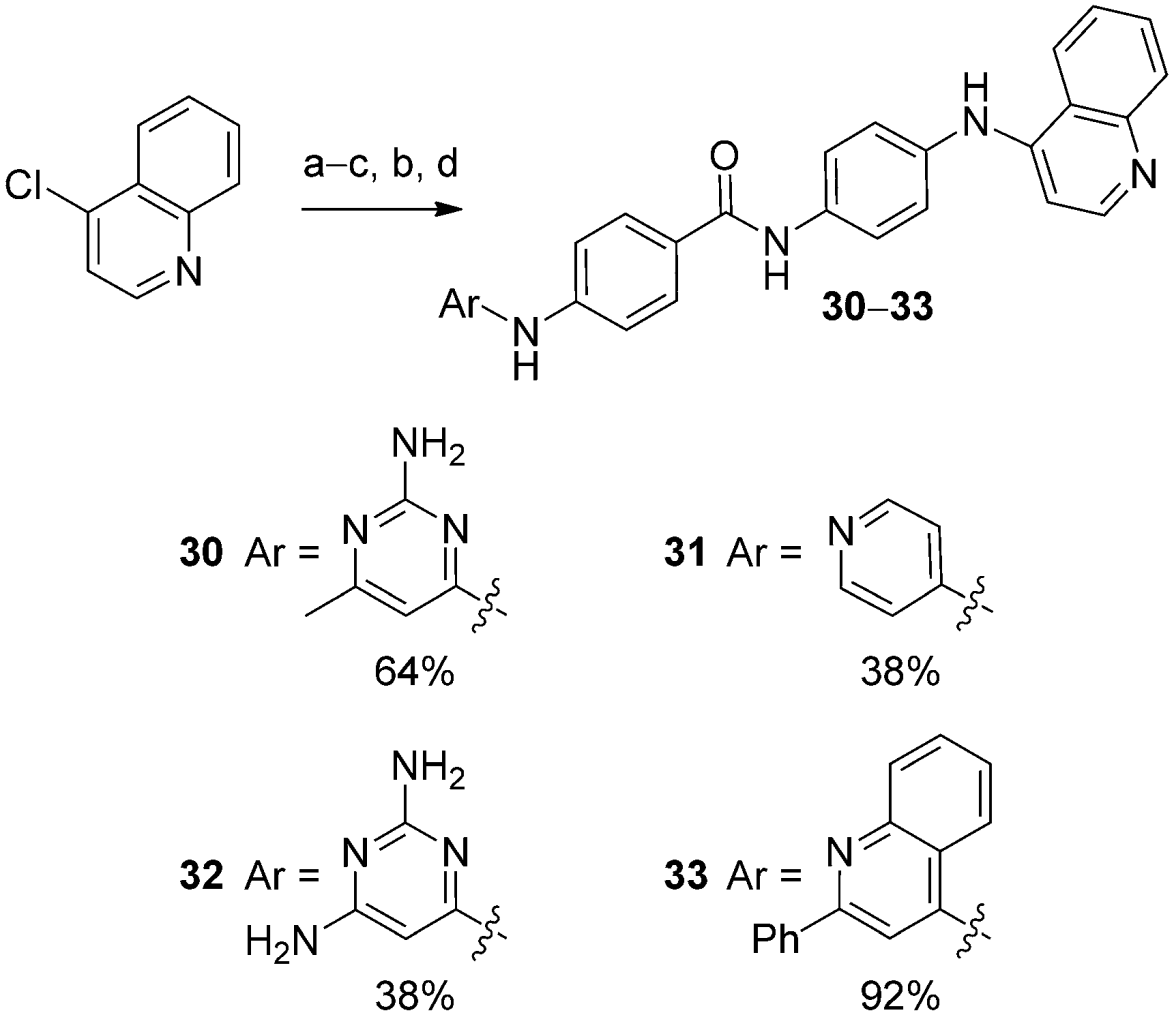
Synthesis of quinoleine derivatives. *Reagents and conditions*: a) 4-nitroaniline, HCl (cat), ethoxyethanol, reflux, 3 h; b) Fe, AcOH, EtOH/H_2_O (2:1), reflux, 15 h; c) 4-nitrobenzoyl chloride, Et_3_N, dioxane, 60 °C, 1 h; d) ArCl, HCl (cat), EtOH/H_2_O/MeOH (1:1:1), reflux, o/n.

After coupling with 4-nitro-aniline, the resulting nitro compound (**26**) was reduced to amino derivative **27** with iron in acetic acid. Amidation with 4-nitro-benzoyl chloride yielded nitro compound **28**, which was similarly reduced to amine **29**. As before, compounds **30**–**33** were prepared by reaction of the corresponding aryl chloride in the presence of a catalytic amount of hydrochloride acid (Scheme 5). Compound **30** is an isomer of SGI-1027 with inversion of the central amide bond of part B. This compound was recently described by Gamage et al. and showed lower efficiency on DNMT1 degradation than SGI-1027.[19] In agreement with these previous findings on DNMT1,[17, 19] methyl-amino pyrimidine **30** was found to be less potent than SGI-1027 against DNMT3A, and its level of activity was comparable to that of diaminopyrimidine **32** (Figure 2 and Table 1). Unexpectedly, compound **32** was difficult to analyze because it did not present a dose–reponse curve but rather a plateau at 50 % inhibition between 32 and 1 μm (data not shown). This could be due to chemical-physical features of the compound that we did not investigate further. Interestingly, compound **31**, containing the quinoline in C and a simplified pyridine cycle in A, was the most potent compound at both 32 and 10 μm, with similar potency than SGI-1027 (Table 1).

### Probing the size of cofactor and substrate pockets

Finally, in order to investigate the size of the cofactor and substrate pockets, we introduced variations to parts A and C, exploring the effect of terminating the molecule with a single ring on both ends (product **36**), with a bicycle (derivatives **33** and **37**) or a tricyclic system (Scheme 5).

**Scheme 6:**
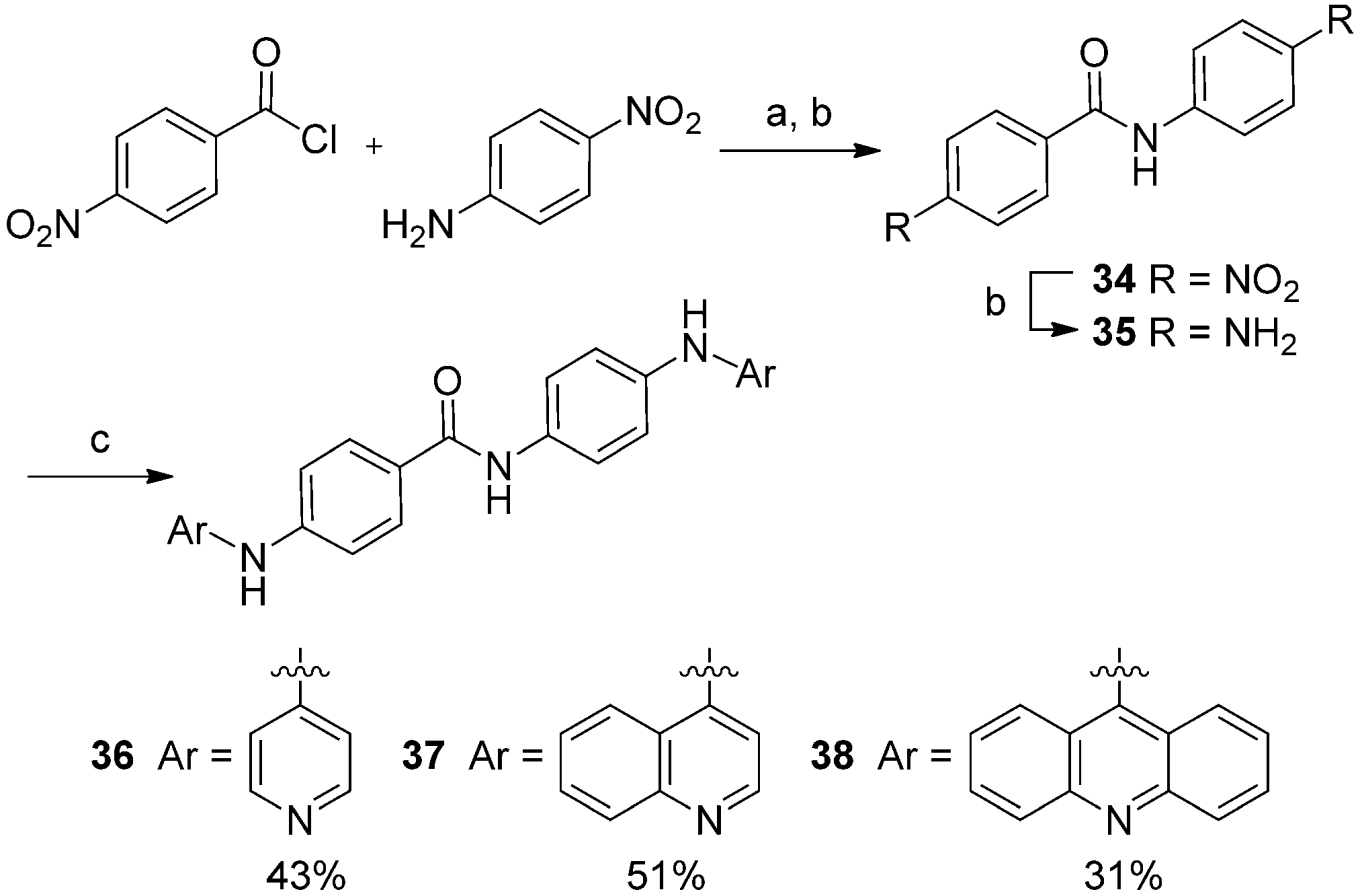
Synthesis of symmetrical compounds. *Reagents and conditions*: a) Et_3_N, dioxane, 60 °C, 1 h; b) Fe, AcOH, EtOH/H_2_O (2:1), reflux, 15 h; c) ArCl, HCl (cat), EtOH/H_2_O/MeOH (1:1:1), reflux, o/n.

Symmetrical compound **36**, terminated with a pyridine moiety, was synthesized according to a simplified synthetic scheme. The central amide part B was easily prepared by reaction between 4-nitrobenzoyl chloride and nitroaniline, followed by simultaneous reduction of the nitro groups to afford compound **35** (Scheme 6). A double arylation with 4-chloropyridine led to **36** in three steps. This simplified pathway offered bis-quinoline **37** (2+2 combination) and bis-acridine **38** (3+3 combination) derivatives.

The inhibitory activity against DNMT3A at 32 and 10 μm confirmed that the best inhibitors remain derivative **31** terminated by a single ring on one side and a two-ring system on the other (Figure 2 and Table 1).

### Biological activities

**Figure 3:**
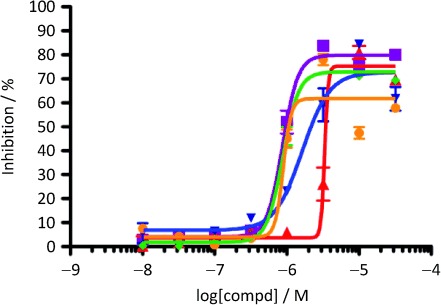
hDNMT3A inhibition curves for SGI-1027 (▪), and compounds 12 (▴), 16 (▾), 31 (⧫) and 32 (•). Data are the mean of triplicate determinations.

The most active compounds against DNMT3A, **12**, **16** and **31** and inactive compound **19** (Figure 2) were further assayed to determine the concentration at which 50 % of efficacy of inhibition is obtained (EC_50_) (Figure 3 and Table 2). We did not further investigate active compound **32** because of its behavior in the dose–reponse experiment discussed above. Compound **31**, the most potent and efficient on hDNMT3A inhibition (EC_50_=0.9 μm, efficacy 90 %), was tested on DNMT1 and on histone lysine methyltransferases G9a inhibition for selectivity. The compound gave an EC_50_ value of 15±3 μm against hDNMT1, comparable to SGI-1027 with an EC_50_ value of 10±1 μm, and a weak inhibition activity on G9A with an IC_50_ value of 28±4 μm, comparable to that of SGI-1027 (IC_50_=59±4 μm).

**Table 2:** Biochemical and biological activity of the most active derivatives. Inhibition (EC_50_) of human DNMT3A catalytic activity, cytotoxicity (EC_50_) against leukemia KG-1 cells, and reactivation of luciferase expression of the CMV-luc construct in KG-1 cells

Compd	EC_50_ [μm]		Fold reactivation^[c]^						
	hDNMT3A^[a]^	KG-1^[b]^	10 μm	5 μm	3.2 μm	1 μm	0.5 μm	0.32 μm	0.1 μm
SGI-1027 (**1**)	0.9 (0.7–1.0)	4.4 (3.2–6.2)	**7.6±1.3**	**15.7±0.2**	**7.1±1.2**	1.4±0.1	1.1±0.1	1.0±0.1	0.9±0.1
**12**	5.0 (1.9–13)	>10	**8.6±0.9**	**2.6±0.1**	1.8±0.1	1.2±0.1	1.1±0.1	1.1±0.1	1.0±0.1
**16**	1.6 (1.0–2.5)	1.3 (1.0–3.2)	**6.1±2.7**	**6.0±2.0**	**5.5±1.3**	1.3±0.1	1.2.±0.1	n.d.	1.0±0.1
**19**	inactive	1.3 (0.9–1.7)	1.0±0.1	1.0±0.1	1.2±0.1	1.0±0.1	1.0±0.1	1.0±0.1	1.0±0.1
**31**	0.9 (0.7–1.0)	0.96 (0.9–1.0)	**3.2±0.5**	**3.9±0.1**	**3.9±0.3**	**2.0±0.2**	1.6±0.3	1.3±0.2	1.1±0.1

[a] Concentration at which 50 % inhibition of enzyme activity was observed; data represent the mean of 3–5 experiments; 95 % confidence intervals are given in parentheses. [b] Concentration at which 50 % inhibition of cellular proliferation was observed; data represent the mean of 3–5 experiments; 95 % confidence intervals are given in parentheses. [c] Fold-induction (reactivation) of the luciferase gene controlled by methylated CMV compared with untreated cells after 24 h of treatment. Data represent the mean±SEM of 2–3 experiments.

The compounds were tested for their ability to reactivate gene expression by use of an integrated luciferase reporter system under the control of a methylated cytomegalovirus (CMV) promoter in leukemia KG-1 cells (CMV-luc assay reported in Table 2), which inhibits its expression. In parallel, the antiproliferative activity of the compounds was tested in KG-1 leukemia cells. In particular, the cytotoxicity in the KG-1 cells of the compounds can interfere with the CMV-luc assay.

The fold induction of the luciferase signal was measured in cells incubated for 24 hours in the presence of the test compounds at the indicated concentrations and normalized to the untreated control cells. Negative control compound **19**, which did not inhibit the enzyme, was also unable to reactivate luciferase expression. Parent compound SGI-1027 gave a 16-fold induction at 5 μm, but at 10 μm, a decrease in the luciferase signal was observed because of its cytotoxicity in the KG-1 cell line (EC_50_=4.4 μm; Table 2). The most potent inhibitor of our series, compound **31**, reactivated luciferase expression starting from 1 μm; however, cytotoxicity in the KG-1 cell line (EC_50_=1 μm; Table 2) hindered the measurement of the luciferase signal at higher concentrations. Active bismonocyclic **12** gave an 8.6-fold induction at 10 μm, a dose that did not affect cell proliferation (Table 2). Compound **16** presented a similar activity profile as compound **31**. Even if the cytotoxicity clearly interefered with the measurement of luciferase induction, we can conclude that all inhibitors induced re-expression of the luciferase gene.

## Conclusions

Three new derivatives of SGI-1027, compounds **12**, **16** and **31**, showed similar DNMT inhibition activity and gene expression induction as the parent compound, associated with proliferation inhibition on KG-1 cells in the micromolar range. Structure–activity relationship studies carried out on three parts of the molecule indicated that the presence of a methylene or carbonyl group as a linker to the quinoline moiety dramatically decreased activity. Second, the effect of the size and nature of the aromatic or heterocycle substituents at the ends of the molecule played an important effect on inhibition: three-ring structures decreased the activity, while two-ring substituents were well tolerated, the best being the combination of a two-ring on one side and a one-ring system on the other. Finally the orientation of the central amide bond was found to have little effect on the activity. These findings bring new insights into the substituents that contribute to the DNMT inhibition activity of this family of compounds for further design of new more potent inhibitors.

## Experimental Section

### Chemistry

Reagents, chemicals and anhydrous solvents were purchased from commercial suppliers and used without purification. Sensitive reactions were performed under an argon atmosphere. NMR spectra were recorded on a Bruker Avance II spectrometer equipped with a ^13^C cryoprobe at 500 MHz for ^1^H and 126 MHz for ^13^C; 2D experiments were performed using standard Bruker programs. High-resolution mass spectrometry (HRMS) was performed on a Bruker MicroTOF spectrometer using electrospray ionization (ESI). Thin-layer chromatography (TLC) was carried out on precoated silica gel 60F_254_ TLC plates (Merck), and spots were visualized by heating after diping in KMnO_4_ solution. Column chromatography was carried out on a Puriflash 430 apparatus (Interchim) equipped with 30 μm porated silica column.

Analytical HPLC was performed on a VWR-Hitachi apparatus ELITE LACHROM. A prepacked C_18_ reverse-phase column (Waters X-Bridge RP-18, 4.6×100 mm, 5 μm) was used for analytical HPLC with a binary gradient elution (solvent A: H_2_O; solvent B: MeCN) and a flow rate of 1 mL min^−1^. Preparative HPLC was performed on an apparatus equipped with a VWR International LaPrep pump P110, a VWR LaPrep P314 dual *λ* absorbance detector, and EZChrom software. A Waters Xbridge RP-18 column (19×250 mm, 10 μm) was used for preparative HPLC with a binary gradient elution (solvent A: H_2_O; solvent B: CH_3_CN) and a flow rate of 25 mL min^−1^, and the UV absorbance was monitored at 250 and 320 nm.

***N***^**4**^**-(4-Nitrophenyl)-6-methylpyrimidine-2,4-diamine (6)**: 4-Nitroaniline **4** (6.00 g, 43.44 mmol) and 2-amino-4-chloro-6-methylpyrimidine **5** (6.24 g, 43.44 mmol) were dissolved in 2-ethoxyethanol (330 mL). A few drops of HCl (35 %) were added, and the solution was heated at reflux for 3 h, and then allowed to cool to RT overnight. The reaction mixture was filtered, and the resulting solid was rinsed with Et_2_O and dried in vacuo to afford compound **6** as a pale yellow solid (8.53 g, 80 %): ^1^H NMR (500 MHz, CD_3_OD): *δ*=2.22 (s, 3 H), 6.04 (d, *J*=0.6 Hz, 1 H), 7.99 (d, *J*=9.2 Hz, 2 H), 8.18 ppm (d, *J*=9.4 Hz, 2 H); ^13^C NMR (126 MHz, CD_3_OD): *δ*=23.3, 97.8, 119.6, 125.8, 148.4, 162.9, 167.3 ppm; MS (ESI): *m*/*z*=246 [*M*+H]^+^.

*N*^**4**^**-(4-Aminophenyl)-6-methylpyrimidine-2,4-diamine (7)**: A refluxing suspension of amine **6** (7.08 g, 28.9 mmol) in EtOH/H_2_O (86 mL/43 mL) was treated with Fe powder (6.44 g, 115.5 mmol) and AcOH (2.6 mL). The resulting dark brown suspension was heated at reflux for 15 h, after which time, the hot mixture was filtered through a pad of Celite. The filtrate was concentrated in vacuo and purified by flash chromatography (CH_2_Cl_2_/MeOH, 100:0→80:20) to afford amine **7** as a brown solid (5.71 g, 92 %): ^1^H NMR (500 MHz, CD_3_OD): *δ*=2.13 (s, 3 H), 5.82 (s, 1 H), 6.73 (d, *J*=8.6 Hz, 2 H), 7.17 ppm (d, *J*=8.6 Hz, 2 H); MS (ESI): *m*/*z*=216 [*M*+H]^+^.

*N***-[4-(2-Amino-6-methylpyrimidin-4-ylamino)phenyl]-4-nitrobenzamide (8)**: 4-Nitrobenzoyl chloride (1.21 g, 6.5 mmol) and Et_3_N (0.99 g, 9.8 mmol) were sequentially added to a solution of amine **7** (1.40 g, 6.5 mmol) in dry dioxane (90 mL). The solution was heated at 60 °C for 1 h, and the reaction mixture was then allowed to cool to RT. Solvent was removed in vacuo, and the residue was purified by flash chromatography on silica gel (CH_2_Cl_2_/MeOH, 100:0→70:30) to give compound **8** (1.63 g, 70 %): ^1^H NMR (500 MHz, [D_6_]DMSO): *δ*=2.08 (s, 3 H), 5.86 (s, 1 H), 6.13 (s, 2 H), 7.66–7.73 (m, 4 H), 8.18 (d, *J*=8.9 Hz, 2 H), 8.37 (d, *J*=8.9 Hz, 2 H), 8.99 (s, 1 H), 10.47 ppm (s, 1 H); ^13^C NMR (126 MHz, [D_6_]DMSO): *δ*=23.5, 94.7, 119.5, 121.0, 123.6, 129.2, 132.5, 132.5, 137.3, 140.8, 149.1, 161.3, 162.9, 163.4, 165.0 ppm; MS (ESI): *m*/*z*=365 [*M*+H]^+^.

***N*****-[4-(2-Amino-6-methylpyrimidin-4-ylamino)phenyl]-4-aminobenzamide (9)**: A refluxing suspension of **8** (1.07 g, 2.9 mmol) in EtOH/H_2_O (13 mL/6.5 mL) was treated with Fe powder (654 mg, 11.7 mmol) and AcOH (1.3 mL). The resulting dark brown suspension was heated at reflux for 15 h, after which time, the resulting mixture was filtered through a pad of silica using 7 n NH_3_ in MeOH. Solvent was removed in vacuo and purification by flash chromatography (CH_2_Cl_2_/MeOH, 100:0→70:30) afforded amine **9** as a brown solid (638 mg, 65 %): ^1^H NMR (500 MHz, [D_6_]DMSO): *δ*=2.07 (s, 3 H), 5.73 (s, 2 H), 5.84 (s, 1 H), 6.09 (s, 2 H), 6.59 (d, *J*=8.6 Hz, 2 H), 7.59–7.63 (m, 4 H), 7.69 (d, *J*=8.6 Hz, 2 H), 8.89 (s, 1 H), 9.67 ppm (s, 1 H); ^13^C NMR (126 MHz, [D_6_]DMSO): *δ*=23.5, 94.7, 112.7, 119.9, 120.8, 121.4, 129.4, 133.8, 136.1, 152.0, 161.5, 163.2, 165.1 ppm; MS (ESI): *m*/*z*=335 [*M*+H]^+^.

***N*****-[4-(2-Amino-6-methylpyrimidin-4-ylamino)phenyl]-4-(pyridin-4-ylamino)benzamide (10)**: A refluxing suspension of amine **9** (100 mg, 0.3 mmol) in MeOH/H_2_O/EtOH (2 mL/2 mL/2 mL) was treated with chloropyridine hydrochloride (34 mg, 0.3 mmol) and one drop of HCl (35 %). The resulting yellow solution was heated at reflux for 22 h, and then was cooled to RT and concentrated in vacuo. Purification by flash chromatography on silica gel (CH_2_Cl_2_/MeOH, 100:0→70:30) gave compound **10** as a yellow solid (98 mg, 80 %): ^1^H NMR (500 MHz, [D_6_]DMSO): *δ*=2.28 (s, 3 H), 6.18 (s, 1 H), 7.29 (d, *J*=6.6 Hz, 2 H), 7.50 (d, *J*=8.7 Hz, 2 H), 7.78–7.83 (m, 4 H), 8.10 (d, *J*=8.6 Hz, 2 H), 8.36 (d, *J*=7.4 Hz, 2 H), 10.40 (s, 1 H), 10.68 (s, 1 H), 11.01 ppm (s, 1 H); ^13^C NMR (126 MHz, [D_6_]DMSO): *δ*=18.4, 97.4, 109.5, 120.9, 121.7, 122.0, 129.5, 131.8, 134.0, 135.8, 140.4, 141.0, 152.3, 155.6, 155.7, 161.4, 164.6 ppm; HRMS (ESI): *m*/*z* [*M*+H]^+^ calcd for C_23_H_22_N_7_O: 412.1886, found: 412.1873.

***N*****-[4-(2-Amino-6-methylpyrimidin-4-ylamino)-phenyl]-4-(pyrimidin-2-ylamino)benzamide (11)**: Compound **11** was synthesized according to the procedure described for **10**, using amine **9** (40 mg (0.12 mmol) and 2-chloropyrimidine (14 mg, 0.12 mmol). Purification by preparative HPLC (H_2_O/CH_3_CN, 90:10→0:100) gave compound **11** as a yellow solid (20 mg, 41 %): ^1^H NMR (500 MHz, [D_6_]DMSO): *δ*=2.08 (s, 3 H), 5.85 (s, 1 H), 6.11 (br s, 2 H), 6.93 (t, *J*=4.8 Hz, 1 H), 7.65 (s, 4 H), 7.91 (s, 4 H), 8.55 (d, *J*=4.7 Hz, 2 H), 8.94 (s, 1 H), 9.98 ppm (d, *J*=9.3 Hz, 2 H); ^13^C NMR (126 MHz, [D_6_]DMSO): *δ*=23.9, 95.0, 113.6, 118.0, 120.1, 121.3, 127.7, 128.8, 133.7, 136.9, 143.9, 158.6, 160.1, 161.8, 163.3, 165.2, 165.3 ppm; HRMS (ESI): *m*/*z* [*M*+H]^+^ calcd for C_22_H_21_N_8_O: 413.1838, found: 413.1825.

***N*****-[4-(2-Amino-6-methylpyrimidin-4-ylamino)phenyl]-*N*-4-(2-amino-6-methylpyrimidin-4-ylamino)benzamide (12)**: Compound **12** was synthesized according to the procedure described for **10**, using amine **9** (40 mg, 0.12 mmol) and 2-amino-4-chloro-6-methylpyrimidine (17 mg, 0.12 mmol). Concentration in vacuo gave compound **12** as a yellow amorphous solid (40 mg, 77 %): ^1^H NMR (500 MHz, [D_6_]DMSO): *δ*= 2.08 (s, 3 H), 2.11 (s, 3 H), 5.87 (s, 1 H), 5.98 (s, 1 H), 6.10 (br s, 2 H), 6.26 (br s, 2 H), 7.66 (s, 4 H), 7.90 (s, 4 H), 9.00 (br s, 1 H), 9.46 (br s, 1 H), 10.00 ppm (br s, 1 H); ^13^C NMR (126 MHz, [D_6_]DMSO): *δ*=23.5, 23.6, 94.6, 95.5, 117.9, 119.6, 120.8, 126.9, 128.4, 133.3, 136.5, 144.1, 161.2, 161.4, 162.9, 162.9, 164.7, 164.9, 165.5 ppm. Filtration of a DMSO solution of **12** through a pad of K_2_CO_3_ gave the free base of **12** as a pale yellow solid quantitatively: ^1^H NMR (500 MHz, [D_6_]DMSO): *δ*=2.08 (s, 3 H), 5.85 (s, 1 H), 6.11 (br s, 2 H), 6.93 (t, *J*=4.8 Hz, 1 H), 7.65 (s, 4 H), 7.91 (s, 4 H), 8.55 (d, *J*=4.7 Hz, 2 H), 8.94 (s, 1 H), 9.98 ppm (d, *J*=9.3 Hz, 2 H); ^13^C NMR (126 MHz, [D_6_]DMSO): *δ*=23.9, 95.0, 113.6, 118.0, 120.1, 121.3, 127.7, 128.8, 133.7, 136.9, 143.9, 158.6, 160.1, 161.8, 163.3, 165.2, 165.3 ppm; HRMS (ESI): *m*/*z* [*M*+H]^+^ calcd for C_22_H_21_N_8_O: 413.1838, found: 413.1825.

***N*****-[4-(2-Amino-6-methylpyrimidin-4-ylamino)phenyl]-4-(4,6-dimethoxy-1,3,5-triazin-2-ylamino)benzamide (13)**: Compound **13** was synthesized according to the procedure described for **10**, using amine **9** (40 mg, 0.12 mmol) and 2-chloro-4,6-dimethoxy-1-3-5-triazine (21 mg, 0.12 mmol). Purification by preparative HPLC (H_2_O/CH_3_CN, 90:10→0:100) gave compound **13** as a white solid (30 mg, 54 %): ^1^H NMR (500 MHz, [D_6_]DMSO): *δ*=10.41 (s, 1 H), 10.03 (s, 1 H), 8.93 (s, 1 H), 7.94 (d, *J*=8.8 Hz, 2 H), 7.88 (d, *J*=8.9 Hz, 2 H), 7.65 (s, 4 H), 6.11 (br s, 2 H), 5.85 (s, 1 H), 3.94 (s, 6 H), 2.07 ppm (s, 3 H); ^13^C NMR (126 MHz, [D_6_]DMSO): *δ*=23.5, 54.7, 94.6, 119.4, 119.6, 120.8, 128.4, 129.0, 133.1, 136.6, 141.9, 161.4, 162.9, 164.6, 164.9, 166.0, 172.0 ppm; HRMS (ESI): *m*/*z* [*M*+H]^+^ calcd for C_23_H_24_N_9_O_3_: 474.2002, found: 474.1996.

***N*****-[4-(2-Amino-6-methylpyrimidin-4-ylamino)phenyl]-4-(quinazolin-4-ylamino)benzamide (14)**: Compound **14** was synthesized according to the procedure described for **10**, using amine **9** (30 mg, 0.09 mmol) and 4-chloroquinazoline (15 mg, 0.09 mmol). Purification by preparative HPLC (H_2_O/CH_3_CN, 90:10→0:100) gave compound **14** as a white solid (25 mg, 73 %): ^1^H NMR (500 MHz, [D_6_]DMSO): *δ*=2.08 (s, 3 H), 5.85 (s, 1 H), 6.13 (br s, 1 H), 7.67–7.72 (m, 4 H), 7.83 (d, *J*=8.1 Hz, 1 H), 7.90 (td, *J*=7.1, 1.3 Hz, 1 H), 8.00 (d, *J*=8.7 Hz, 2 H), 8.08 (d, *J*=8.7 Hz, 2 H), 8.61 (d, *J*=8.4 Hz, 1 H), 8.69 (s, 1 H), 8.96 (s, 1 H), 10.03 (br s, 1 H), 10.10 ppm (s, 1 H); ^13^C NMR (126 MHz, [D_6_]DMSO): *δ*=23.6, 94.6, 115.4, 119.7, 120.9, 121.2, 123.1, 126.6, 128.0, 128.2, 129.6, 133.2, 133.3, 136.7, 142.4, 149.8, 154.4, 157.6, 161.4, 163.0, 164.7, 165.0 ppm; HRMS (ESI): *m*/*z* [*M*+H]^+^ calcd for C_26_H_23_N_8_O: 463.1995, found: 463.1970.

***N*****-[4-(2-Amino-6-methylpyrimidin-4-ylamino)phenyl]-4-(7-fluoroquinazolin-4-ylamino)benzamide (15)**: Compound **15** was synthesized according to the procedure described for **10**, using amine **9** (50 mg, 0.15 mmol) and 4-chloro-7-fluoroquinazoline (25 mg, 0.15 mmol). Purification by preparative HPLC (H_2_O/CH_3_CN, 90:10→0:100) gave compound **15** as a white solid (12 mg, 18 %): ^1^H NMR (500 MHz, [D_6_]DMSO): *δ*=2.08 (s, 3 H), 5.85 (s, 1 H), 6.13 (s, 2 H), 7.58–7.62 (m, 2 H), 7.67 (s, 4 H), 8.03 (d, *J*=15.5 Hz, 4 H), 8.68–8.72 (m, 2 H), 8.96 (s, 1 H), 10.11 ppm (s, 1 H); ^13^C NMR (126 MHz, [D_6_]DMSO): *δ*=23.6, 94.6, 111.9 (d, *J*_C–F_=20.3 Hz), 112.5, 116.1 (d, *J*_C–F_=24.4 Hz), 119.7, 120.9, 121.3, 126.6 (d, *J*_C–F_=10.6 Hz), 128.2, 129.8, 133.2, 136.7, 142.1, 151.9 (d, *J*_C–F_=13.5 Hz), 155.6, 157.5, 161.4, 162.9, 164.7, 164.9, 164.8 ppm (d, *J*_C–F_=251.8 Hz); HRMS (ESI): *m*/*z* [*M*+H]^+^ calcd for C_26_H_22_FN_8_O: 481.1901, found: 481.1884.

***N*****-[4-(2-Amino-6-methylpyrimidin-4-ylamino)phenyl]-4-(2-phenylquinolin-4-ylamino)benzamide (16)**: Compound **16** was synthesized according to the procedure described for **10**, using amine **9** (30 mg, 0.09 mmol) and 4-chloro-2-phenylquinoline (22 mg, 0.09 mmol). Purification by preparative HPLC (H_2_O/CH_3_CN, 90:10→0:100) gave compound **16** as a white solid (16 mg, 33 %): ^1^H NMR (500 MHz, [D_6_]DMSO): *δ*=2.08 (s, 3 H), 5.85 (s, 1 H), 6.13 (br s, 2 H), 7.45–7.48 (m, 1 H), 7.50–7.53 (m, 2 H), 7.56–7.60 (m, 3 H), 7.65–7.69 (m, 4 H), 7.72 (s, 1 H), 7.69 (td, *J*=6.9, 1.3 Hz, 1 H), 8.00 (dd, *J*=8.4, 1.0 Hz, 1 H), 8.04 (d, *J*=8.7 Hz, 2 H), 8.09–8.11 (m, 2 H), 8.39 (d, *J*=8.4 Hz, 1 H), 8.95 (s, 1 H), 9.34 (s, 1 H), 10.10 ppm (s, 1 H); ^13^C NMR (126 MHz, [D_6_]DMSO): *δ*=24.0, 95.0, 101.2, 120.0, 120.1, 120.1, 121.2, 122.6, 125.4, 127.5, 129.1, 129.2, 129.7, 129.7, 130.0, 130.4, 133.6, 137.0, 139.9, 147.7, 149.5, 157.1, 161.8, 163.4, 164.9, 165.3 ppm; HRMS (ESI): *m*/*z* [*M*+H]^+^ calcd for C_33_H_28_N_7_O: 538.2355, found: 538.2348.

***N*****-[4-(2-Amino-6-methylpyrimidin-4-ylamino)phenyl]-4-(7*H*-purin-6-ylamino)benzamide (17)**: Compound **17** was synthesized according to the procedure described for **10**, using amine **9** (50 mg, 0.15 mmol) and 6-chloropurine (23 mg, 0.15 mmol). Purification by flash chromatography on silica gel (CH_2_Cl_2_/MeOH, 100:0→60:40) gave compound **17** as a white solid (3 mg, 5 %): ^1^H NMR (500 MHz, [D_6_]DMSO): *δ*=2.08 (s, 3 H), 5.85 (s, 1 H), 6.10 (s, 1 H), 7.65 (d, *J*=2.8 Hz, 4 H), 7.91 (d, *J*=8.8 Hz, 2 H), 8.16 (d, *J*=8.8 Hz, 2 H), 8.35 (s, 1 H), 8.92 (s, 1 H), 9.96 ppm (s, 1 H); MS (ESI): *m*/*z*=453.2 [*M*+H]^+^.

***N*****-[4-(2-Amino-6-methylpyrimidin-4-ylamino)-phenyl]-4-(acrindin-9-ylamino)benzamide (18)**: Compound **18** was synthesized according to the procedure described for **10**, using amine **9** (60 mg, 0.18 mmol) and 9-chloroacridine (38 mg, 0.18 mmol). Purification by preparative HPLC (H_2_O/CH_3_CN, 90:10→0:100) gave compound **18** as a white solid (51 mg, 55 %): ^1^H NMR (500 MHz, [D_6_]DMSO): *δ*=2.07 (s, 3 H), 5.85 (s, 1 H), 6.11 (br s, 2 H), 6.88 (d, *J*=8.5 Hz, 2 H), 6.90–6.99 (m, 2 H), 7.35 (d, *J*=7.7 Hz, 2 H), 7.50 (t, *J*=9.6 Hz, 1 H), 7.70–7.60 (m, 5 H), 7.85–7.79 (m, 2 H), 7.97 (d, *J*=8.5 Hz, 2 H), 8.15 (t, *J*=7.4 Hz, 2 H), 8.92 (br s, 1 H), 10.01 (br, 1 H), 11.05 ppm (br s, 1 H); ^13^C NMR (126 MHz, [D_6_]DMSO): *δ*=23.5, 94.6, 114.8, 116.8, 117.7, 119.7, 120.8, 121.1, 124.3, 125.1, 127.2, 129.1, 129.6, 129.7, 130.4, 131.7, 133.4, 136.5, 150.8, 157.1, 161.4, 162.9, 164.7, 164.9 ppm; HRMS (ESI): *m*/*z* [*M*+H]^+^ calcd for C_22_H_21_N_8_O: 512.2193, found: 512.2198.

***N*****-[4-(2-Amino-6-methylpyrimidin-4-ylamino)phenyl]-4-(isonicotinamide)benzamide (19)**: A suspension of isonicotinoyl chloride hydrochloride (50 mg, 0.28 mmol) in pyridine (0.5 mL) was cooled to 0 °C, and a solution of amine **9** (94 mg, 0.28 mmol) in pyridine (1.5 mL) was added dropwise. The reaction was stirred at RT overnight. The mixture was then filtered, and the isolated solid was purified by flash chromatography on silica gel (CH_2_Cl_2_/MeOH, 100:0→70:30) to give compound **19** as a white solid (38 mg, 30 %): ^1^H NMR (500 MHz, [D_6_]DMSO): *δ*=2.07 (s, 3 H), 5.88 (s, 1 H), 6.11 (s, 1 H), 7.68 (s, 4 H), 7.99 (d, *J*=5.2 Hz, 6 H), 8.77 (d, *J*=5.4 Hz, 2 H), 9.14 (s, 1 H), 10.30 ppm (s, 1 H); HRMS (ESI): *m*/*z* [*M*+H]^+^ calcd for C_24_H_22_N_7_O_2_: 440.1835, found: 440.1817.

***N*****-[4-(2-Amino-6-methylpyrimidin-4-ylamino)phenyl]-4-(nicotinamide)benzamide (20)**. Compound **20** was synthesized according to the procedure described for **19**, using nicotinoyl chloride hydrochloride (100 mg, 0.56 mmol) and amine **9** (188 mg, 0.56 mmol). Purification by flash chromatography on silica gel (CH_2_Cl_2_/MeOH, 100:0→70:30) gave compound **20** as a light brown solid (40 mg, 30 %): ^1^H NMR (500 MHz, [D_6_]DMSO): *δ*=2.27 (s, 3 H), 6.13 (s, 1 H), 7.60 (dd, *J*=7.8.9, 4.9 Hz, 1 H), 7.92–8.03 (m, 4 H), 7.95 (d, *J*=8.8 Hz, 2 H), 8.01 (d, 8.8 Hz, 2 H), 8.34 (d, *J*=8.1 Hz, 1 H), 8.79 (dd, *J*=4.7, 1.4 Hz, 1 H), 9.14 (d, *J*=1.8 Hz, 1 H), 10.27 (s, 1 H), 10.52 (s, 1 H), 10.74 (s, 1 H), 12.49 ppm (s, 1 H); ^13^C NMR (126 MHz, [D_6_]DMSO): *δ*=18.4, 97.1, 112.6, 119.5, 120.7, 123.6, 128.6, 129.8, 130.3, 133.7, 135.7, 141.9, 148.9, 152.3, 155.8, 161.4, 164.4, 164.8 ppm; HRMS (ESI): *m*/*z* [*M*+H]^+^ calcd for C_24_H_22_N_7_O_2_: 440.1835, found: 440.1825.

***N*****-[4-(2-Amino-6-methylpyrimidin-4-ylamino)phenyl]-4-(2-hydroxynicotinamide)benzamide (21)**: Compound **21** was synthesized according to the procedure described for **19**, upon use of 2-hydroxy-3-nicotinic acid (90 mg, 0.65 mmol) and amine **9** (72 mg, 0.22 mmol). The resulting mixture was purified by flash chromatography on silica gel (CH_2_Cl_2_/MeOH, 100:0→60:40) and by preparative HPLC (H_2_O/CH_3_CN, 90:10→0:100) to give compound **21** as a light yellow solid (20 mg, 20 %): ^1^H NMR (500 MHz, [D_6_]DMSO): *δ*=2.08 (s, 3 H), 5.85 (s, 1 H), 6.12 (s, 2 H), 6.53 (dd, *J*=7.2, 5.9 Hz, 1 H), 7.82 (d, *J*=8.8 Hz, 2 H), 7.66 (s, 4 H), 7.87 (dd, *J*=5.9, 2.3 Hz, 1 H), 7.97 (d, *J*=8.8 Hz, 1 H), 8.38 (dd, *J*=7.2, 2.3 Hz, 1 H), 8.95 (s, 1 H), 10.08 (s, 1 H), 13.04 ppm (s, 1 H); ^13^C NMR (126 MHz, [D_6_]DMSO): *δ*=23.6, 94.6, 107.1, 118.2, 118.9, 119.7, 120.9, 128.8, 129.5, 133.2, 136.6, 141.6, 143.2, 143.4, 161.4, 163.0, 164.5, 164.6, 164.9 ppm; HRMS (ESI): *m*/*z* [*M*+H]^+^ calcd for C_24_H_22_N_7_O_3_: 456.1784, found: 456.1753.

***N*****-[4-(2-Amino-6-methylpyrimidin-4-ylamino)-phenyl]-4-(quinoline-4-carboxylamide)benzamide (22)**. Compound **22** was synthesized according to the procedure described for **19**, using quinoline carboxylic acid (85 mg, 0.49 mmol) and amine **9** (82 mg, 0.25 mmol). Purification by flash chromatography (CH_2_Cl_2_/MeOH, 100:0→70:30) gave compound **22** as a light yellow solid (27 mg, 23 %): ^1^H NMR (500 MHz, [D_6_]DMSO): *δ*=2.12 (s, 3 H), 5.91 (s, 1 H), 7.86 (td, *J*=6.7, 1.3 Hz, 1 H), 7.63 (d, *J*=4.3 Hz, 1 H), 7.77 (d, *J*=4.3 Hz, 1 H), 7.86 (td, *J*=6.9, 1.4 Hz, 1 H), 7.93 (d, *J*=8.7 Hz, 2 H), 8.00 (d, *J*=8.8 Hz, 2 H) 8.03 (d, *J*=8.7 Hz, 2 H), 8.15 (t, *J*=8.9 Hz, 2 H), 8.66 (dd, *J*=8.6, 1.1 Hz, 1 H), 9.06 (d, *J*=4.3 Hz, 1 H), 9.25 (br s, 1 H), 10.16 (s, 1 H), 11.08 ppm (s, 1 H); ^13^C NMR (126 MHz, [D_6_]DMSO): *δ*=22.5, 95.1, 119.3, 119.4, 120.0, 124.0, 125.2, 125.3, 126.5, 127.1, 128.7, 129.6, 130.2, 130.4, 133.7, 136.2, 141.6, 141.7, 148.0, 148.4, 150.4, 150.5, 161.5, 164.6, 165.7 ppm; MS (ESI): *m*/*z*=490 [*M*+H]^+^.

***N*****-[4-(2-Amino-6-methylpyrimidin-4-ylamino)phenyl]-4-(methylamino)benzamide (23) and**­ ***N*****-[4-(2-Amino-6-methylpyrimidin-4-ylamino)phenyl]-4-(pyridin-3-ylmethylamino)benzamide (24)**: Pyridinium chlorochromate (PCC; 395 mg, 1.83 mmol) was added to a solution of 4-pyridinemethanol (100 mg, 0.92 mmol) in CH_2_Cl_2_ (5 mL), and the mixture was stirred for 90 min at RT. The resulting mixture was diluted with CH_2_Cl_2_ (25 mL) and then washed with water (2×25 mL) and brine (1×25 mL). The organic phase was dried over Na_2_SO_4_, filtered and concentrated in vacuo. The residue was dissolved in MeOH (2 mL), treated with Et_3_N (38 mg, 0.38 mmol) and amine **9** (40 mg, 0.12 mmol), and the solution was stirred at RT for 3.5 h. AcOH (0.053 mL) and NaBH_3_CN (30 mg) were then added, and the solution was stirred at RT overnight. The resulting mixture was purified by preparative HPLC (H_2_O/CH_3_CN, 90:10→0:100) to give compound **23** as a light brown solid (9 mg, 17 %) and compound **24** as a pale brown solid (1.1 mg, 3 %). Compound **23**: ^1^H NMR (500 MHz, [D_6_]DMSO): *δ*=2.07 (s,3 H), 2.73 (d, *J*=5.0 Hz, 3 H), 5.83 (s, 1 H), 6.09 (s, 2 H), 6.31 (q, *J*=4.9 Hz, 1 H), 6.67 (d, *J*=8.8 Hz, 2 H), 7.62 (s, 4 H), 8.60 (d, *J*=8.8 Hz,1 H), 8.89 (s, 1 H), 9.70 ppm (s, 1 H); ^13^C NMR (126 MHz, [D_6_]DMSO): *δ*=23.5, 29.3, 94.5, 110.4, 119.7, 120.6, 121.1, 133.7, 136.1, 152.5,161.4, 162.9, 169.2, 164.8, 165.0 ppm; HRMS (ESI): *m*/*z* [*M*+H]^+^ calcd for C_19_H_21_N_6_O: 371.1771, found: 371.1589. Compound **24**: ^1^H NMR (500 MHz, [D_6_]DMSO): *δ*=2.09 (s, 3 H), 4.42 (d, *J*=6.3 Hz, 2 H), 5.84 (s, 1 H), 6.10 (s, 2 H), 6.62 (d, *J*=8.8 Hz, 2 H), 7.03 (t, *J*=6.2 Hz, 1 H), 7.35 (d, *J*=5.8 Hz, 2 H), 7.61 (s, 4 H), 7.72 (d, *J*=8.8 Hz, 2 H), 8.51 (d, 6.0 Hz, 2 H), 8.91 (s, 1 H), 9.71 ppm (s, 1 H); ^13^C NMR (126 MHz, [D_6_]DMSO): *δ*=23.9, 45.3, 94.9, 111.7, 120.1, 121.1, 122.4, 122.7, 129.6, 134.0, 136.5, 149.5, 150.0, 151.4, 161.8, 163.8, 165.3, 165.4 ppm; HRMS (ESI): *m*/*z* [*M*+Na]^+^ calcd for C_24_H_23_N_7_ONa: 448.1862, found: 448.1844.

***N*****-[4-(2-Amino-6-methylpyrimidin-4-ylamino)-phenyl]-4-(pyridin-4-ylmethylamino)benzamide (25)**: MnO_2_ (2.10 g, 24.2 mmol) was added to a solution of 3-pyridinemethanol (330 mg, 3.0 mmol) in CH_2_Cl_2_ (5 mL), and the reaction mixture was stirred at RT overnight. The mixture was filtered through a pad of celite, and solvent was removed in vacuo. The residue was dissolved in MeOH (5 mL), treated with Et_3_N (100 mg, 0.99 mmol) and amine **9** (80 mg, 0.24 mmol), and then stirred at RT overnight. AcOH (0.150 mL) and NaBH_3_CN (145 mg) were then added, and the solution was stirred at RT for 3 h. The resulting mixture was purified by preparative HPLC (H_2_O/CH_3_CN, 90:10→0:100) to give compound **25** as a light yellow solid (98 mg, 96 %): ^1^H NMR (500 MHz, [D_6_]DMSO): *δ*=2.06 (s, 3 H), 4.39 (d, *J*=6.1 Hz, 2 H), 5.84 (s, 1 H), 6.10 (s, 2 H), 6.65 (d, *J*=8.8 Hz, 2 H), 6.95 (t, *J*=6.2 Hz, 1 H), 7.36 (dd, *J*=7.8, 4.8 Hz, 1 H), 7.60 (br s, 4 H), 7.72 (d, *J*=8.8 Hz, 2 H), 7.75 (dt, *J*=7.8 Hz, 1.8 Hz, 1 H), 8.45 (dd, *J*=4.8, 1.7 Hz, 1 H), 8.60 (d, *J*=1.9 Hz, 1 H), 9.71 (s, 1 H), 8.90 ppm (s, 1 H); ^13^C NMR (126 MHz, [D_6_]DMSO): *δ*=23.6, 43.6, 94.5, 111.3, 119.7, 120.6, 121.9, 123.6, 129.2, 133.7, 135.1, 135.1, 136.2, 148.1, 149.0, 151.0, 161.4, 162.9, 164.9, 165.0 ppm; HRMS (ESI): *m*/*z* [*M*+H]^+^ calcd for C_24_H_24_N_7_O_1_: 426.2042, found: 426.2037.

***N*****-(4-Nitrophenyl)quinolin-4-amine (26)**: Compound **26** was synthesized according to the procedure described for **6**, using 4-nitroaniline (4.13 g, 29.90 mmol) and 4-chloroquinoline (4.89 g, 29.90 mmol). The reaction mixture was filtered, and the resulting solid was rinsed with Et_2_O and dried in vacuo to afford compound **26** as a pale brown solid (3.73 g, 47 %): ^1^H NMR (500 MHz, [D_6_]DMSO): *δ*=7.26 (d, *J*=6.8 Hz, 1 H), 7.80 (d, *J*=8.9 Hz, 2 H), 7.87 (td, *J*=7.1, 1.3 Hz, 1 H), 8.08 (td, *J*=7.1, 1.3 Hz, 1 H), 8.15 (d, *J*=8.5 Hz, 1 H), 8.40 (d, *J*=9.1 Hz, 2 H), 8.70 (d, *J*=6.8 Hz, 1 H), 8.84 (d, *J*=8.2 Hz, 1 H), 11.23 (s, 1 H), 15.03 ppm (s, 1 H); ^13^C NMR (126 MHz, [D_6_]DMSO): *δ*=102.2, 118.6, 121.0, 124.4, 125.8, 128.0, 134.6, 139.0, 144.0, 144.6, 145.2, 154.3 ppm; HRMS (ESI): *m*/*z* [*M*+H]^+^ calcd for C_24_H_24_N_7_O: 266.0924, found: 266.0928.

***N*****-(Quinolin-4-yl)benzene-1,4-diamine (27)**: Compound **27** was synthesized according to the procedure described for **7**, using **26** (3.73 g, 14.07 mmol). The filtrate was concentrated in vacuo and purified by flash chromatography (CH_2_Cl_2_/MeOH, 100:0→70:30) to give amine **27** as a brown solid (2.93 g, 89 %): ^1^H NMR (500 MHz, [D_6_]DMSO): *δ*=5.33 (br s, 2 H), 6.56 (d, *J*=6.7 Hz, 1 H), 6.69 (d, *J*=8.6 Hz, 2 H), 7.06 (d, *J*=8.6 Hz, 2 H), 7.63–7.70 (m, 1 H), 7.86–7.91 (m, 1 H), 7.94 (d, *J*=8.3 Hz, 1 H), 8.39 (d, *J*=6.6 Hz, 1 H), 8.59 (d, *J*=8.5 Hz, 1 H), 10.11 ppm (br s, 1 H); ^13^C NMR (126 MHz, [D_6_]DMSO): *δ*=99.5, 114.5, 117.4, 122.6, 122.9, 125.5, 126.1, 126.7, 132.5, 141.2, 144.6, 148.1, 154.1 ppm; MS (ESI): *m*/*z*=236 [*M*+H]^+^.

***N*****-[4-(Quinolin-4-ylamino)phenyl]-4-nitrobenzamide (28)**. Compound **28** was synthesized according to the procedure described for **8**, using 4-nitrobenzoyl chloride (1.37 g, 7.40 mmol) and amine **27** (1.74 g, 7.40 mmol). The filtrate was concentrated in vacuo and purified by flash chromatography (CH_2_Cl_2_/MeOH, 100:0→70:30) to give compound **28** as a yellow solid (2.60 g, 93 %): ^1^H NMR (500 MHz, [D_6_]DMSO): *δ*=6.84 (d, *J*=6.3 Hz, 1 H), 7.47 (d, *J*=8.7 Hz, 2 H), 7.69–7.75 (m, 2 H), 7.88–7.91 (m, 1 H), 7.93–7.98 (m, 3 H), 8.23 (d, *J*=8.8 Hz, 2 H), 8.40 (d, *J*=8.8 Hz, 2 H), 8.50 (d, *J*=6.3 Hz, 1 H), 8.60 (d, *J*=8.5 Hz, 1 H), 8.60 (d, *J*=8.5 Hz, 1 H), 10.76 ppm (s, 1 H); MS (ESI): *m*/*z*=385 [*M*+H]^+^.

***N*****-[4-(Quinolin-4-ylamino)phenyl]-4-aminobenzamide (29)**: Compound **29** was synthesized according to the procedure described for **7**, using **28** (1.40 g, 3.64 mmol). The filtrate was concentrated in vacuo and purified by flash chromatography (CH_2_Cl_2_/MeOH, 100:0→80:20) to give amine **29** as a yellow solid (326 mg, 25 %): ^1^H NMR (500 MHz, [D_6_]DMSO): *δ*=5.77 (s, 1 H), 6.61 (d, *J*=8.7 Hz, 2 H), 6.78 (d, *J*=6.1 Hz, 1 H), 7.36 (d, *J*=8.8 Hz, 2 H), 7.65 (t, *J*=8.9 Hz, 1 H), 7.73 (d, *J*=8.7 Hz, 2 H), 7.84–7.81 (m, 1 H), 7.88 (d, *J*=8.8 Hz, 1 H), 7.91 (br d, *J*=8.3 Hz, 1 H), 8.46 (d, *J*=6.1 Hz, 1 H), 8.63 (br d, *J*=7.9 Hz, 1 H), 9.89 ppm (br s, 1 H); MS (ESI): *m*/*z*=355 [*M*+H]^+^.

***N*****-[4-(Quinolin-4-ylamino)phenyl]-4-(2-amino-6-methylpyrimidin-4-ylamino)benzamide (30)**: Compound **30** was synthesized according to the procedure described for **10**, using amine **29** (70 mg, 0.20 mmol) and 2-amino-4-chloro-6-methylpyrimidine (43 mg, 0.30 mmol). Purification by preparative HPLC (H_2_O/CH_3_CN, 90:10→0:100) gave compound **30** as a white solid (58 mg, 64 %): ^1^H NMR (500 MHz, [D_6_]DMSO): *δ*=5.95 (s, 1 H), 6.27 (br s, 2 H), 6.83 (d, *J*=5.3 Hz, 1 H), 7.34 (d, *J*=8.8 Hz, 1 H), 7.52 (td, *J*=6.9, 1.2 Hz, 1 H), 7.69 (td, *J*=6.8, 1.3 Hz, 1 H), 7.83 (d, *J*=8.9 Hz, 2 H), 7.86 (dd, *J*=8.6, 1.0 Hz, 1 H), 7.94–7.88 (m, 4 H), 8.39 (br d, *J*=8.0 Hz, 1 H), 8.43 (d, *J*=5.3 Hz, 1 H), 8.92 (br s, 1 H), 9.34 (br s, 1 H), 10.12 ppm (br s, 1 H); ^13^C NMR (126 MHz, [D_6_]DMSO): *δ*=23.6, 95.5, 100.9, 117.9, 119.5, 121.3, 122.0, 123.5, 124.5, 126.7, 128.5, 129.2, 129.2, 135.6, 135.8, 144.2, 148.2, 148.9, 150.7, 161.1, 162.9, 164.9, 165.6 ppm; HRMS (ESI): *m*/*z* [*M*+H]^+^ calcd for C_27_H_24_N_7_O: 462.2037, found: 462.2034.

***N*****-[4-(Quinolin-4-ylamino)phenyl]-4-(pyridin-4-ylamino)benzamide (31)**: Compound **31** was synthesized according to the procedure described for **10**, using amine **29** (55 mg, 0.16 mmol) and 4-chloropyridine hydrochloride (47 mg, 0.31 mmol). Purification by preparative HPLC (H_2_O/CH_3_CN, 90:10→0:100) gave compound **31** as a yellow solid (25 mg, 38 %): ^1^H NMR (500 MHz, [D_6_]DMSO): *δ*=6.84 (d, *J*=5.5 Hz, 1 H), 7.05 (d, *J*=6.4 Hz, 2 H), 7.31 (d, *J*=8.8 Hz, 2 H), 7.35 (d, *J*=7.0 Hz, 2 H), 7.52 (td, *J*=7.0, 1.2 Hz, 1 H), 7.69 (td, *J*=6.9, 1.2 Hz, 1 H), 7.84 (d, *J*=8.9 Hz, 2 H), 7.86 (d, *J*=8.4 Hz, 1 H), 7.97 (d, *J*=8.7 Hz, 2 H), 8.28 (d, *J*=6.3 Hz, 2 H), 8.39 (d, *J*=8.5 Hz, 1 H), 8.43 (d, *J*=5.4 Hz, 1 H), 8.93 (s, 1 H), 9.18 (s, 1 H), 10.17 ppm (s, 1 H); ^13^C NMR (126 MHz, [D_6_]DMSO): *δ*=100.8, 110.2, 117.6, 119.4, 121.2, 121.9, 123.3, 124.4, 127.6, 129.0, 129.1, 129.1, 135.5, 135.6, 143.8, 148.1, 148.8, 148.8, 150.2, 157.6, 164.6 ppm; HRMS (ESI): *m*/*z* [*M*+H]^+^ calcd for C_27_H_22_N_5_O: 432.1824, found: 432.1815.

***N*****-[4-(Quinolin-4-ylamino)phenyl]-4-(2,6-diaminopyrimidin-4-ylamino)benzamide (32)**: Compound **32** was synthesized according to the procedure described for **10**, using amine **29** (80 mg, 0.24 mmol) and 2,6-diamino-4-chloropyrimidine (32 mg, 0.24 mmol). Purification by preparative HPLC (H_2_O/CH_3_CN, 90:10→0:100) gave compound **32** as a white solid (39 mg, 38 %): ^1^H NMR (500 MHz, [D_6_]DMSO): *δ*=5.26 (s, 1 H), 5.76 (br s, 2 H), 5.92 (br s, 2 H), 6.82 (d, *J*=5.3 Hz, 1 H), 6.84 (d, *J*=5.3 Hz, 1 H), 7.22 (d, *J*=5.1 Hz, 1 H), 7.34 (d, *J*=8.8 Hz, 1 H), 7.52 (td, *J*=6.9, 1.4 Hz, 1 H), 7.69 (td, *J*=6.9, 1.3 Hz, 1 H), 7.79 (d, *J*=8.9 Hz, 1 H), 7.88–7.82 (m, 5 H), 8.39 (br d, *J*=8.6 Hz, 1 H), 8.43 (d, *J*=5.3 Hz, 1 H), 8.92 (br s, 2 H), 10.06 ppm (br s, 1 H); ^13^C NMR (126 MHz, [D_6_]DMSO): *δ*=77.5, 100.9, 117.4, 117.7, 119.5, 121.3, 122.0, 123.5, 124.5, 125.6, 128.4, 129.2, 135.5, 135.9, 145.2, 148.2, 148.9, 150.7, 161.0, 162.9, 164.8, 165.0 ppm; HRMS (ESI): *m*/*z* [*M*+H]^+^ calcd for C_26_H_23_N_8_O_1_: 463.1989, found: 463.1987.

***N*****-[4-(Quinolin-4-ylamino)phenyl]-4-(2-phenylquinolin-4-ylamino)benzamide (33)**: Compound **33** was synthesized according to the procedure described for **10**, using amine **29** (80 mg, 0.24 mmol) and 4-chloro-2-phenylquinoline (81 mg, 0.34 mmol). Purification by preparative HPLC (H_2_O/CH_3_CN, 90:10→0:100) gave compound **33** as a white solid (116 mg, 92 %): ^1^H NMR (500 MHz, [D_6_]DMSO): *δ*=6.85 (d, *J*=5.2 Hz, 1 H), 7.36 (d, *J*=8.8 Hz, 2 H), 7.54–7.46 (m, 4 H), 7.62–7.56 (m, 3 H), 7.69 (td, *J*=6.9, 1.3 Hz, 1 H), 7.37 (s, 1 H), 7.77 (td, *J*=6.9, 1.3 Hz, 1 H), 7.85–7.88 (m, 3 H), 8.01 (dd, *J*=8.5, 0.9 Hz, 1 H), 8.06 (d, *J*=8.7 Hz, 2 H), 8.09–8.11 (m, 2 H), 8.40 (br d, *J*=8.5 Hz, 2 H), 8.44 (d, *J*=5.2 Hz, 1 H), 8.94 (br s, 1 H), 9.34 (br s, 1 H), 10.15 ppm (br s, 1 H); ^13^C NMR (126 MHz, [D_6_]DMSO): *δ*=101.4, 120.0, 120.1, 121.7, 122.5, 122.7, 123.9, 124.9, 125.5, 125.6, 127.5, 128.9, 129.2, 129.6, 129.7, 129.8, 130.0, 136.1, 136.2, 139.6, 139.9, 144.9, 147.7, 148.6, 149.3, 149.5, 151.1, 157.1, 165.2 ppm; HRMS (ESI): *m*/*z* [*M*+H]^+^ calcd for C_37_H_28_N_5_O: 558.2288, found: 558.2292.

***N*****-(4-Nitrophenyl)-4-nitrobenzamide (34)**: Compound **34** was synthesized according to the procedure described for **8**, using 4-nitrobenzoyl chloride (6.72 mg, 3.62 mmol) and 4-nitroaniline (5.0 g, 3.62 mmol). Filtration of the precipitate and drying in vacuo gave compound **34** as a yellow solid (10.29 mg, 99 %): ^1^H NMR (500 MHz, [D_6_]DMSO): *δ*=8.09 (d, *J*=8.3 Hz, 2 H), 8.24 (d, *J*=8.9 Hz, 2 H), 8.29 (d, *J*=9.2 Hz, 2 H), 8.39 (d, *J*=8.8 Hz, 2 H), 11.20 ppm (br s, 1 H); ^13^C NMR (126 MHz, [D_6_]DMSO): *δ*=120.1, 123.6, 124.8, 129.6, 139.8, 142.8, 145.0, 149.4, 164.7 ppm.

***N*****-(4-Aminophenyl)-4-aminobenzamide (35)**: Compound **35** was synthesized according to the procedure described for **7**, using compound **34** (5.10 g, 17.8 mmol). Filtration over a pad of silica and concentration in vacuo gave diamine **35** as a brown solid (1.08 g, 27 %): ^1^H NMR (500 MHz, [D_6_]DMSO): *δ*=5.72 (br s, 4 H), 6.57 (d, *J*=8.6 Hz, 2 H), 6.61 (d, *J*=8.7 Hz, 2 H), 7.39 (d, *J*=8.8 Hz, 2 H), 7.67 (d, *J*=8.6 Hz, 2 H), 9.45 ppm (br s, 1 H); ^13^C NMR (126 MHz, [D_6_]DMSO): *δ*=112.9, 116.5, 121.8, 122.3, 129.6, 131.9, 140.4, 152.2, 165.2 ppm; HRMS (ESI): *m*/*z* [*M*+Na]^+^ calcd for C_13_H_13_N_3_ONa: 250.0951, found: 250.0951.

***N*****-[4-(Pyridin-4-ylamino)phenyl]-4-(pyridin-4-ylamino)benzamide (36)**: Compound **36** was synthesized according to the procedure described for **10**, using amine **35** (80 mg, 0.35 mmol) and chloropyridine hydrochloride (110 mg, 0.74 mmol). Purification by HPLC (H_2_O/CH_3_CN, 90:10→0:100) gave compound **36** as a white solid (57 mg, 43 %): ^1^H NMR (500 MHz, [D_6_]DMSO): *δ*=6.84 (d, *J*=6.5 Hz, 2 H), 7.04 (d, *J*=6.5 Hz, 2 H), 7.18 (d, *J*=8.9 Hz, 2 H), 7.30 (d, *J*=8.6 Hz, 2 H), 7.75 (d, *J*=8.9 Hz, 2 H), 7.96 (d, *J*=8.6 Hz, 2 H), 8.16 (d, *J*=6.5 Hz, 2 H), 8.28 (d, *J*=6.7 Hz, 2 H), 8.72 (br s, 1 H), 9.18 (br s, 1 H), 10.10 ppm (br s, 1 H); ^13^C NMR (126 MHz, [D_6_]DMSO): *δ*=108.8, 110.3, 117.8, 120.9, 121.4, 127.7, 129.2, 134.6, 135.8, 143.9, 148.9, 150.1, 150.4, 150.5, 164.6 ppm; HRMS (ESI): *m*/*z* [*M*+H]^+^ calcd for C_23_H_20_N_5_O: 382.1662, found: 382.1661.

***N*****-[4-(Quinolin-4-ylamino)phenyl]-4-(quinolin-4-ylamino)benzamide (37)**: Compound **37** was synthesized according to the procedure described for **10**, using amine **35** (80 mg, 0.35 mmol) and 4-chloroquinoline (173 mg, 1.06 mmol). Purification by HPLC (H_2_O/CH_3_CN, 90:10→0:100) gave compound **37** as a white solid (86 mg, 51 %): ^1^H NMR (500 MHz, [D_6_]DMSO): *δ*=6.84 (d, *J*=5.3 Hz, 1 H), 7.22 (d, *J*=5.1 Hz, 1 H), 7.36 (d, *J*=8.8 Hz, 1 H), 7.50 (d, *J*=8.5 Hz, 1 H), 7.53 (td, *J*=7.1 Hz, 1.3 Hz, 1 H), 7.58 (td, *J*=7.1 Hz, 1.0 Hz, 1 H), 7.69 (td, *J*=6.8 Hz, 1.2 Hz, 1 H), 7.74 (td, *J*=6.7 Hz, 1.3 Hz, 1 H), 7.88–7.83 (m, 3 H), 7.93 (d, *J*=8.3 Hz, 1 H), 8.03 (d, *J*=8.7 Hz, 2 H), 8.38 (d, *J*=8.7 Hz, 1 H), 8.40 (d, *J*=8.5 Hz, 1 H), 8.44 (d, *J*=5.2 Hz, 1 H), 8.59 (d, *J*=5.1 Hz, 1 H), 8.97 (br s, 1 H), 9.27 (br s, 1 H), 10.23 ppm (br s, 1 H); ^13^C NMR (126 MHz, [D_6_]DMSO): *δ*=101.0, 103.8, 119.5, 119.7, 120.5, 121.4, 122.4, 122.4, 123.5, 124.6, 125.1, 128.6, 129.1, 129.2, 129.3, 129.4, 129.6, 135.7, 135.7, 144.4, 146.4, 148.3, 148.8, 149.1, 150.6, 150.8, 164.8 ppm; HRMS (ESI): *m*/*z* [*M*+H]^+^ calcd for C_31_H_24_N_5_O: 482.1975, found: 482.1974.

***N*****-[4-(Acridin-9-ylamino)phenyl]-4-(acridin-9-ylamino)benzamide (38)**: Compound **38** was synthesized according to the procedure described for **10**, using amine **35** (50 mg, 0.22 mmol) and 9-chloroacridine (118 mg, 0.55 mmol). Purification by HPLC (H_2_O/CH_3_CN, 90:10→0:100) gave compound **38** as a white solid (40 mg, 31 %): ^1^H NMR (500 MHz, [D_6_]DMSO): *δ*=6.74 (d, *J*=8.8 Hz, 2 H), 6.78 (d, *J*=8.5 Hz, 2 H), 6.97–6.92 (m, 4 H), 7.51–7.41 (m, 8 H), 7.71 (d, *J*=8.7 Hz, 2 H), 7.88 (d, *J*=8.5 Hz, 4 H), 7.92 (d, *J*=8.3 Hz, 2 H), 9.91 ppm (br s, 1 H); ^13^C NMR (126 MHz, [D_6_]DMSO): *δ*=117.4, 118.0, 118.5, 119.6, 120.3, 121.7, 121.8, 124.8, 126.9, 129.3, 130.4, 130.9, 133.5, 145.4, 151.5, 157.3, 164.9 ppm; HRMS (ESI): *m*/*z* [*M*+H]^+^ calcd for C_39_H_28_N_5_O: 582.2288, found: 582.2281.

### Docking analysis

The structure of *Haemophilus haemolyticus* cytosine-5 DNA methyltransferase (MHhaI C5 DNMT) was taken from the Protein Data Bank (PDB: 2HR1[16]). Discovery Studio 3.0 (Accelrys Inc., San Diego, CA, USA) to prepare the enzyme structures; alternate conformations were removed and incomplete chains with missing residues and hydrogen atoms were added. The ligands were docked under standard conditions using SYBYl-X 1.3 software (surflex-dock V2) from Tripos L.P. (St. Louis, MO, USA). The images were prepared with Benchware 3D Explorer, also from Tripos.

### Biological evaluation

*DNMT3A assay*: DNMT3A enzyme inhibition was adapted from the restriction-based fluorescence assay protocol described by Ceccaldi et al.[20] Briefly, a 5′-labelled biotin oligonucleotide was hybridized to its complementary strand labelled with 6-carboxyfluorescein at the 3′-end and transferred into a 384-well microplate (black Optiplates; PerkinElmer) pre-coated with avidin. The duplex contains a unique CpG site overlapping with a restriction site of a methylation-sensitive restriction enzyme. The human C-terminal DNMT3A (a.a. 623-908), produced as described in Ref. [20], was added to each well (200 ng/well) and mixed with the chemical compounds at the desired concentration and freshly prepared AdoMet (20 μm final concentration) to start the reaction in a total volume of 50 μL. After 1 h incubation at 37 °C, each well was washed three times with phosphate-buffered saline (PBS) containing 0.05 % Tween-20 and NaCl (500 mm) and three more times with phosphate-buffered saline Tween-20 (PBST). Specific fluorescence signals were detected with the methylation-sensitive restriction enzyme HpyCH4IV (New England Biolabs, Ipswich, MA, USA) as described, and measured on a PerkinElmer Envision detector. The percent inhibition was calculated according to Equation (1), where X is the signal determined in the absence of the inhibitor and Y is the signal obtained in the presence of the inhibitor.


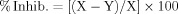
(1)

The ligand concentration at which 50 % inhibition of enyme activity is observed (EC_50_) was determined by analysis of a concentration range of the test compounds in triplicates. Nonlinear regression fittings with sigmoidal dose–response (variable slope) were performed with Prism 4.03 (GraphPad Software, Inc., La Jolla, CA, USA).

*DNMT1 and G9A assay*: His-DNMT1 (182 kDa, human) was cloned, expressed and purified as described by Lee et al.[21] The assays were performed as described in the literature.[22] The reaction was performed in a total reaction volume of 10 μL in low-volume nonbinding surface (NBSTM) 384-well microplates (Corning Inc.), containing test compound (up to 1 % DMSO), 1 μm of a *S*-adenosyl-l-methionine (SAM)/[methyl-3H]SAM (3 TBq mmol^−1^, PerkinElmer) mix in a ratio of 3:1 (isotopic dilution 1*:3), 0.3 μm of biotinylated hemimethylated DNA duplex (5′-GAT*mC*GC*mC*GATG*mC*G*mC*GAAT*mC*G*mC*GAT*mC*GATG*mC*GAT-3′ and BIOT-5′-ATCGCATCGATCGCGATTCGCGCATCGGCGATC-3′), and 90 nm of DNMT1 in methylation buffer (20 mm HEPES (pH 7.2), 1 mm EDTA, 50 mm KCl, 25 μg mL^−1^ bovine serum albumin). The reaction was incubated at 37 °C for 2 h, then an aliquot (8 μL) was transferred into a streptavidin 96-well scintillant-coated FlashPlate (PerkinElmer) containing 20 μm *S*-adenosyl-l-homocysteine (SAH; 190 μL) in 50 mM Tris-HCl (pH 7.4). The FlashPlate was agitated at RT for 1 h, washed three times with 200 μL of 0.05 % TweenR-20 in 50 mm Tris-HCl (pH 7.4), and read in 200 μL of 50 mm Tris-HCl (pH 7.4) on TopCount NXT (PerkinElmer).

*CMV-luc reexpression*: KG-1 cells, stably transfected with the firefly luciferase (luc+ from pGL3 by Promega) reporter gene under the control of the cytomegalovirus (CMV) promoter (from pEGFP-N1 by Clontech Laboratories Inc.) partially methylated (50 %), was seeded at 20 000 cells per well in a 96-well plate. After 24 h incubation in the presence of the test compound or solvant (DMSO), the induction of the promoter was measured by quantification of luciferase with the Brite-lite assay system (PerkinElmer) according to the manufacturer′s protocol. The luminescence was measured on an EnVision multilabel plate reader (PerkinElmer), and the data are expressed as the fold induction as compared with the DMSO control. The mean of three experiments and the standard error is reported.

*Antiproliferative activity*: KG-1 human leukemia cells were obtained from the American Type Culture Collection (ATCC, Manassas, VA, USA) and cultivated in RPMI-1640 medium containing 4-(2-hydroxyethyl)-1-piperazineethanesulfonic acid (HEPES) and l-glutamine (BE12-115F, Lonza, France) supplemented with 15 % fetal calf serum (Lonza, France), at 37 °C and under 5 % CO_2_. Cells (2×10^4^) were seeded at day 0 in a 96-well plate. Solutions of test compound, stored at −20 °C as a 10^−2^ m stock solution in 100 % DMSO, were freshly prepared by dilution on day 1 in RPMI-1640 medium. Cells were treated with test compound solutions at a dose range from 3.2 nm to 10 μm. Treatment was repeated on days 2 and 3, and on day 4, cell viability was assessed using the ATPLite kit (ATPlite 1step Luminescence Assay System, ref: 3016739, PerkinElmer), following the manufacturer′s instructions. Raw data were analyzed with Prism 4.03 to generate EC_50_ values corresponding to the compound concentration required to cause a 50 % decrease in cell viability as compared with untreated controls. The values presented are the mean of at least two independent experiments.

## Supporting Information

As a service to our authors and readers, this journal provides supporting information supplied by the authors. Such materials are peer reviewed and may be re-organized for online delivery, but are not copy-edited or typeset. Technical support issues arising from supporting information (other than missing files) should be addressed to the authors.

miscellaneous_information
