# Supplementary material for: Design, Synthesis and Biological Evaluation of 4-Amino-N-(4-aminophenyl)benzamide Analogues of Quinoline-Based SGI-1027 as Inhibitors of DNA Methylation
Source: ChemMedChem. 2014 Feb 13;9(3):590–601. doi: 10.1002/cmdc.201300420 (PMC4506529; doi:10.1002/cmdc.201300420)
Supplement: Supplementary file 1 — miscellaneous_information [file cmdc0009-0590-sd1.pdf]

CHEMMEDCHEM
Chemistry & Drug Discovery

Supporting Information

© Copyright Wiley-VCH Verlag GmbH & Co. KGaA, 69451 Weinheim, 2014

Design, Synthesis and Biological Evaluation of 4-Amino-*N*-(4-aminophenyl)benzamide Analogues of Quinoline-Based SGI-1027 as Inhibitors of DNA Methylation

Elodie Rilova,[a] Alexandre Erdmann,[a] Christina Gros,[a] Véronique Masson,[a] Yannick Aussagues,[a] Valérie Poughon-Cassabois,[a] Arumugam Rajavelu,[b] Albert Jeltsch,[b] Yoann Menon,[a] Natacha Novosad,[a] Jean-Marc Gregoire,[a] Stéphane Vispé,[a] Philippe Schambel,[c] Fréderic Ausseil,[a] François Sautel,[a] Paola B. Arimondo,*[a] and Frédéric Cantagrel*[a]

cmdc_201300420_sm_miscellaneous_information.pdf

Supporting Information

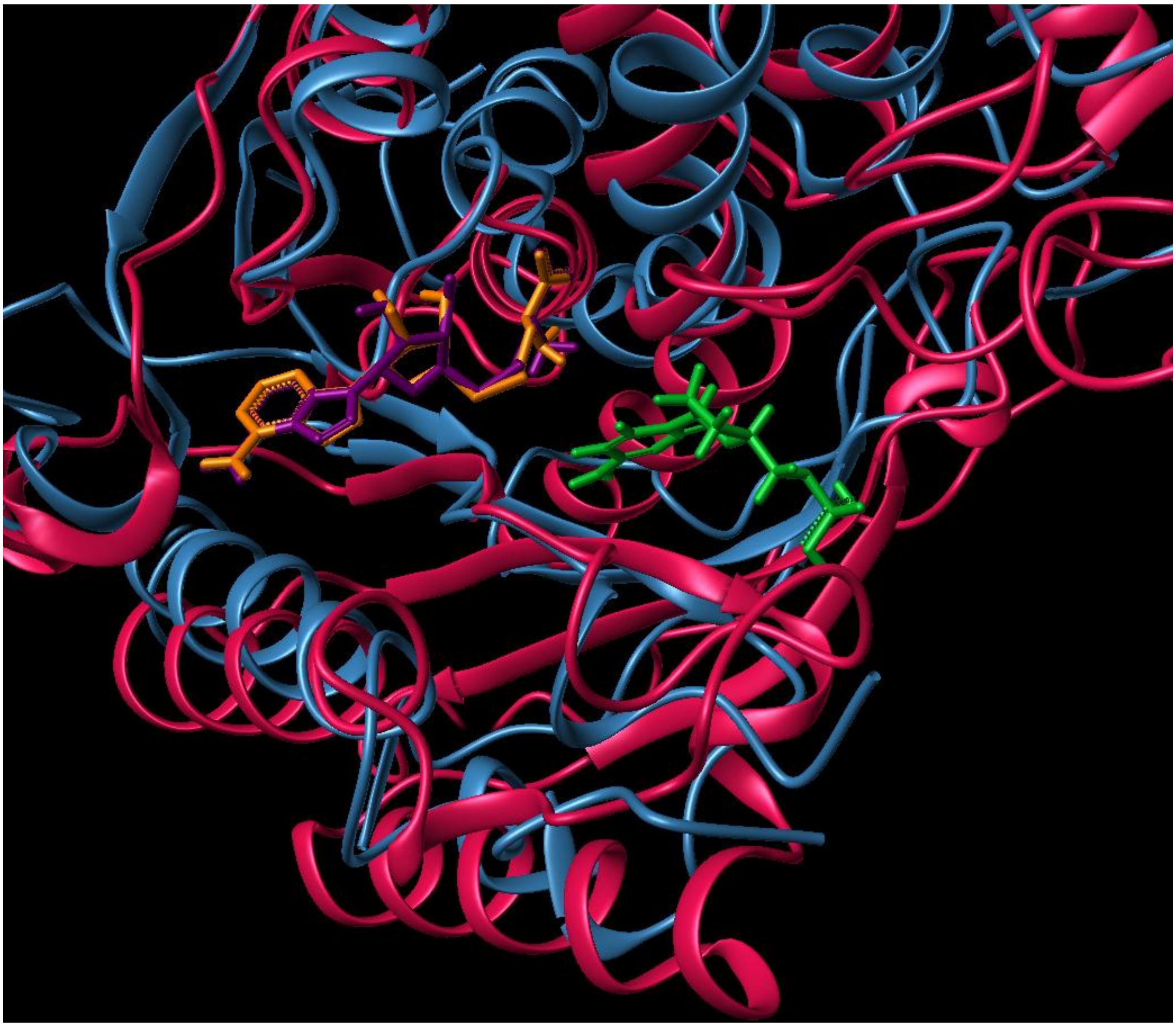

Figure S1. Superposition of the catalytic pocket of DNA methyltransferase of M.Hha I (PDB: 2HR1, in pink) and catalytic Dnmt3A (PDB: 2QRV in cyan). The cofactors, AdoHcy (in orange in M.Hha I and in purple in Dnmt3A) on the left and deoxycytidine (in green in M.Hha I) on the right, are shown.
